# The autophagy-related proteins FvAtg4 and FvAtg8 are involved in virulence and fumonisin biosynthesis in *Fusarium verticillioides*

**DOI:** 10.1080/21505594.2022.2066611

**Published:** 2022-04-20

**Authors:** Yujie Wang, Xin Liu, Yujiao Xu, Yiying Gu, Xinyue Zhang, Mengxuan Zhang, Wen Wen, Yin-Won Lee, Jianrong Shi, Sherif Ramzy Mohamed, Amira A. Goda, Huijun Wu, Xuewen Gao, Qin Gu

**Affiliations:** a Department of Plant Pathology, College of Plant Protection, Nanjing Agricultural University/Key Laboratory of Monitoring and Management of Crop Diseases and Pest Insects, Ministry of Education, Nanjing, Jiangsu, China; b Safety and Quality, Ministry of Agriculture and Rural Affairs/Key Laboratory for Agro-product Safety Risk Evaluation (Nanjing), Ministry of Agriculture and Rural Affairs/Collaborative Innovation Center for Modern Grain Circulation and Safety/Institute of Food Safety and Nutrition, Jiangsu Academy of Agricultural SciencesJiangsu Key Laboratory for Food Quality and Safety-State Key Laboratory Cultivation Base, Ministry of Science and Technology/Key Laboratory for Control Technology and Standard for Agro-product, Nanjing, Jiangsu, China; c School of Food and Biological Engineering, Jiangsu University, Zhenjiang, Jiangsu, China; d School of Agricultural Biotechnology, Seoul National University, Seoul, Republic of Korea; e Food Toxicology and Contaminants Department, National Research Centre, Giza, Egypt

**Keywords:** Autophagy, Fusarium verticillioides, virulence, fumonisin biosynthesis

## Abstract

Autophagy is the main intracellular degradation system by which cytoplasmic materials are transported to and degraded in the vacuole/lysosome of eukaryotic cells, and it also controls cellular differentiation and virulence in a variety of filamentous fungi. However, the contribution of the autophagic pathway to fungal development and pathogenicity in the important maize pathogen and mycotoxigenic fungus *Fusarium verticillioides* is still unknown. In this study, we characterized two autophagy-related proteins, FvAtg4 and FvAtg8. The *F. verticillioides* deletion mutants Δ*FvAtg4* and Δ*FvAtg8* were impaired in autophagosome formation, aerial hyphal formation, sexual growth, lipid turnover, pigmentation and fungal virulence. Interestingly, Δ*FvAtg4* and Δ*FvAtg8* were defective in fumonisin B1 (FB1) synthesis, which may have resulted from decreased intracellular levels of alanine in the mutants. Our results indicate that FvAtg4 and FvAtg8 contribute to *F. verticillioides* pathogenicity by regulating the autophagic pathway to control lipid turnover, fumonisin biosynthesis, and pigmentation during its infectious cycle.

## Introduction

*Fusarium verticillioides*, a causal agent of ear and stock rot in maize (*Zea mays*), has emerged as a model pathogen to study the interaction between fungi and plants [1,2]. The importance of understanding the biology of the mycotoxigenic fungus *F. verticillioides*, as well as its diverse microbial and plant host interactions, is critical given its threat to maize, one of the world’s most valuable agricultural crops. During infection, *F. verticillioides* produces mycotoxin contamination in grains, which pose a risk to economic returns, as well as to human and animal health and food security.

The main classes of fumonisin (FB) are FB1, FB2 and FB3. To date, 28 related compounds have been identified as FBs, including FB1, which has been classified as a Group 2B carcinogen; it has high toxigenicity and is usually found in food [3]. Approximately 70% of natural FB pollution is related to these compounds. Despite the economic impacts and food safety concerns caused by *F. verticillioides*, the molecular mechanisms underlying pathogenesis and FB biosynthesis in this fungus are not well understood, which limits the establishment of highly efficient strategies to manage this disease. Therefore, a deep understanding of fungal biology and host-pathogen interactions in *F. verticillioides* is urgently needed.

Autophagy is an intracellular process required for the turnover of proteins and organelles and a degradation system that transports cytoplasmic material to lysosomes/vacuoles during development and in response to nutrient stress in eukaryotic cells [4–6]. Autophagy has attracted medical research interest during the past decade due to its connection with cancer, neurodegenerative disease and various human developmental processes [7,8]. Autophagy has been reported to have an important role in asexual and sexual development, cell stress management, nutritional starvation stress, and pathogenic development in filamentous fungi including *Magnaporthe oryzae*, *Podospora anserine*, *Aspergillus oryzae* and *Fusarium oxysporum* [9,10]. Furthermore, it has been suggested that autophagy may be involved in lipid turnover, a process that is associated with fungal pathogenic processes in *F. graminearum* and *M. oryzae* [11–14]. Importantly, recent studies have also revealed the possible involvement of autophagy in mycotoxin biosynthesis in *F. graminearum*, which extends our knowledge of autophagy in filamentous fungi [14,15]. Nevertheless, the specific roles of autophagy in the development and virulence of most filamentous fungi, especially *F. verticillioides*, have not been completely described.

In *Saccharomyces cerevisiae*, genetic screening has led to the identification of over 30 autophagy-related (*ATG*) genes [16]. Atg8, an ubiquitin-like protein, was found to form a conjugate with the lipid phosphatidylethanolamine (PE), and this conjugate (Atg8-PE) is required for the formation of double-membrane autophagosomes [17–19]. The cysteine protease Atg4 was found to be crucial for the cleavage of the C-terminus of Atg8. This truncated form of Atg8 is the substrate for the conjugation of Atg8 to PE [20,21]. The well-studied autophagic pathway in budding yeast has provided an excellent road map for understanding autophagy in eukaryotes. Using the yeast model, the functions of Atg8 have been identified in filamentous fungi including *M. oryzae*, *Ustilago maydis*, *A. oryzae*, *F. graminearum* and *P. anserine*. Deletion of the *ATG8* gene resulted in defects in autophagy, virulence, and cellular growth and differentiation in various filamentous fungi [14,22–24]. Interaction between MoAtg4 and MoAtg8 is required for pathogenesis in *M. oryzae* [25]. However, the roles of Atg4 and Atg8 in most pathogenic fungi remain unknown, and how Atg4 and Atg8 function together to regulate virulence is only partially understood to date.

Similarly, no experimental evidence has emerged to explain the functions of the autophagy-related proteins Atg4 and Atg8 in *F. verticillioides*. Here, we characterized orthologs of these autophagy-related proteins (named FvAtg4 and FvAtg8) and elucidated how autophagy affects fungal growth, development, virulence and secondary metabolism. Our results demonstrated that FvAtg4 functions positively with FvAtg8 and that both are involved in the formation of aerial mycelium and in lipid turnover, which explains the regulation of *F. verticillioides* virulence by the FvAtg4 and FvAtg8 proteins. Moreover, our study showed that FB1 biosynthesis was impaired in the *FvATG4* and *FvATG8* deletion mutants, which may result from decreased levels of intracellular alanine in both mutants.

## Materials and methods

### Fungal strains and culture conditions

The wild-type strain *Fusarium verticillioides* 7600 and its corresponding mutants used in this study were cultured at 25°C on complete medium (CM) (per liter: 10 g glucose, 2 g peptone, 1 g yeast extract, 1 g casamino acids, nitrate salts, trace elements, 0.01% vitamins, pH adjusted to 6.5, solidified with 10 g agar), potato dextrose agar (PDA) (per liter: 200 g potato, 20 g glucose, 10 g agar), and minimal medium (MM) (10 mM K_2_HPO_4_, 10 mM KH_2_PO_4_, 4 mM (NH_4_)_2_SO_4_, 2.5 mM NaCl, 2 mM MgSO_4_, 0.45 mM CaCl_2_, 9 mM FeSO_4_, 10 mM glucose, 1% agar, pH 6.9) for hyphal growth tests according to a previous publication [14]. To detect conidiation in each strain, we transferred six mycelial plugs (5-mm diameter) from 3-d-old colonies into a 50-ml flask containing 20 ml of liquid CMC medium (per liter: 0.5 g (NH_4_)_2_SO_4_, 1 g NaNO_3_, 1 g KH_2_PO_3_, 0.5 g MgSO_4_.7 H_2_O, 15 g Carboxymethylcellulose sodium) according to a previous publication [26]. The flasks were incubated at 25°C for 4 d in a shaker (180 rpm). The experiment was repeated independently three times. Conidial numbers were measured with a hemacytometer. For the sexual development of each strain, sexual mating assays were performed according to previously described protocols [26]. The experiment was repeated independently three times.

### Complementation of *S.* *cerevisiae* mutants

The wild-type *S. cerevisiae* strain BY4741, *ATG4* deletion mutant (Δ*Atg4*), and *ATG8* deletion mutant (Δ*Atg8*) were obtained from EUROSCARF (http://web.uni-frankfurt.de/fb15/mikro/euroscarf/). The full-length *FvATG4* and *FvATG8* cDNAs were cloned into the pYES2 vector and were then transformed into the yeast Δ*Atg4* and Δ*Atg8* mutants of strain BY4741, respectively. *S. cerevisiae* transformants were screened on synthetic medium lacking uracil (Clontech, Laboratories, Inc.). BY4741, Δ*Atg4* and Δ*Atg8* were also transformed with the empty pYES2 vector as controls.

### Generation of gene deletion and complemented strains

A double-joint PCR approach was used to generate gene deletion constructs using the primers listed in Table S1, which were then transformed into protoplasts of *F. verticillioides* 7600 using a protocol described previously [27]. FvAtg4 and FvAtg8 deletion mutants were generated using a homologous recombination strategy. Among 108 hygromycin-resistant transformants, four FvATG4 deletion mutants (Δ*FvAtg4*) and three FvATG8 deletion mutants (Δ*FvAtg8*) were identified by diagnostic PCR with the primer pairs A5 + A6 and A11 + A12, respectively (Table S1). Complementation was performed by reintroduction of full-length FvATG4 and FvATG8 into their deletion mutants (Δ*FvAtg4* and Δ*FvAtg8*, respectively). The complemented strains Δ*FvAtg4*-C and Δ*FvAtg8*-C were identified by PCR analysis with the primer pairs A5 + A6 and A11 + A12, respectively. To analyze the relationship between FvAtg4 and FvAtg8, the double deletion mutant strain Δ*FvAtg4-8* was generated and identified by diagnostic PCR with the primer pair A11 + A12. Double deletion mutants (Ag4-bk3, Ag8-bk3, Ag4-bk1, and Ag8-bk1) were obtained using the same strategy. The fungal strains were stored as a conidial suspension in 30% glycerol at −70 °C.

### Analysis of pigments from *F.* *verticillioides*

Melanin pigments from each *F. verticillioides* mutant were extracted using a previously reported method [28]. The extracted pigments and synthetic melanin (Sigma-Aldrich, MO, USA) were then analyzed by using Fourier transform infrared (FT-IR) spectroscopy (Thermo Fisher Scientific, Warsaw, Poland) following the protocols described by Drewnowska et al [29]. To detect the production of bikaverin, each *F. verticillioides* strain was cultured at 25°C in PDB medium for 10 d. A 100 mL culture of each strain was extracted three times with ethyl acetate acidified with 1 mL of 25% HCl [30]. The samples were then analyzed on an HPLC–MS system (G2-XS QTof, Waters) using a protocol previously described [30].

### Staining and microscopic observations

To construct the FvAtg4-GFP fusion cassette, *FvATG4* containing the promoter region and the open-reading fragment (without stop codon) was amplified from genomic DNA of wild-type 7600 using the primer pair *FvATG4*-GFP-F+*FvATG4*-GFP-R (Table S1), the resulting PCR products were co-transformed with *Xho* I-digested PYF11(*NEO* selective marker) into the yeast strain XK1–25 as described previously [31]. The FvAtg4-GFP fusion plasmid was extracted with the Yeast Plasmid Kit (Solarbio, Beijing, China) was then transferred into *E. coli* DH5α. Finally, the recombined plasmid obtained from DH5α was transformed into the wild type 7600 or Δ*FvAtg4*. The GFP-FvAtg8, *FvATG4* promoter-GFP or *FvATG8* promoter-GFP fusion vector were fused and transformed using the same strategy. The strains used in this study are shown in Table 1. Autophagic bodies in each strain were examined using a Leica TCS SP5 imaging system and freshly harvested mycelia from minimum medium (MM) lacking a nitrogen source in the presence of phenylmethanesulfonyl fluoride (PMSF). To observe GFP fluorescence, fresh mycelia and conidia were examined under a Zeiss LSM780 confocal microscope (Carl Zeiss AG, Germany). To observe vacuoles, freshly harvested mycelia were stained with 7-amino-4-chloromethylocoumarin (CMAC, Molecular Probes, USA). Transmission electron microscopy observations were carried out as described by Jiang et al [32].

**Table 1.** *Fusarium verticillioides* strains used in this study

Strains	Genotype description	Reference
7600	Wild type (*MAT1-1*) of *F. verticillioides*	(Xu and Leslie, 1996)
7598	Wild type (MAT1–2) of *F. verticillioides*	(Xu and Leslie, 1996)
ΔFvATG4	*FvATG4* deletion mutant of 7600	This study
ΔFvATG8	*FvATG8* deletion mutant of 7600	This study
ΔFvATG4-8	*FvATG4 FvATG8* double deletion	This study
ΔFvATG4-C	*FvATG4* complemented transformant of Δ*FvAtg4*	This study
ΔFvATG8-C	*FvATG8* complemented transformant of Δ*FvAtg8*	This study
AG4-N1	FvAtg4-GFP transformant of Δ*FvAtg4*	This study
AG8-N2	GFP-FvAtg8 transformant of Δ*FvAtg8*	This study
AG4-R8	GFP-FvAtg8 transformant of Δ*FvAtg4*	This study
AG8-R4	FvAtg4-GFP transformant of Δ*FvAtg8*	This study
D56	GFP-FvAtg8&FvAtg4-RFP transformant of 7600	This study
W-AG8	GFP-FvAtg8 transformant of 7600	This study
AG4-W2	*FvATG4* promoter-GFP transformant of 7600	This study
AG8-W4	*FvATG8* promoter-GFP transformant of 7600	This study
Ag4-bk3	*FvBIK3* deletion mutant of Δ*FvAtg4*	This study
Ag8-bk3	*FvBIK3* deletion mutant of Δ*FvAtg8*	This study
Ag4-bk1	*FvBIK1* deletion mutant of Δ*FvAtg4*	This study
Ag8-bk1	*FvBIK1* deletion mutant of Δ*FvAtg8*	This study

### Pathogenicity assays

For the pathogenicity tests, 10 μL conidial suspensions (10^6^ conidia/mL) of each strain were injected into a 12-week-old maize stalk that was wounded with a hole punch, and 10 μL of sterilized water was injected into maize stalks as a control. Each strain had six replicates. Plants were kept in a growth chamber under 80% humidity at 25 ± 2°C After inoculation for 15 d, the extent of rot in each infected maize plant was examined after splitting the stalks longitudinally. The ability of the mutant strains to infect maize kernels was also determined according to a previously reported method [31].

### Cell wall-degrading enzyme (CWDE) activity assays

CWDE activity was compared between the wild-type 7600 strain and the mutants (Δ*FvAtg4*, Δ*FvAtg8*, and Δ*FvAtg4-8*) by culturing the strains in liquid MM medium containing 1% broken maize stems. Total endoglucanase and xylanolytic enzyme activities were then analyzed by measuring the release of sugars from the substrates. Briefly, 270 μL of the substrate solution (1% carboxymethylcellulose sodium salt or 1% oat spelt xylan) was added and mixed with 30 μL of culture supernatant. The endoglucanase and xylanolytic activities were analyzed by measuring the reducing sugars released from the substrates using a protocol previously described [33].

### Measurement of intracellular triglycerides and alanine levels

Triglycerides were extracted from mycelia of each strain cultured in liquid MM for 3 d. The amount of triglyceride (per mg protein) was determined with the Triglyceride Quantification Kit (Pplygen, Beijing, China). For the measurement of the intracellular alanine levels in the wild-type 7600, Δ*FvAtg4*, Δ*FvAtg8*, and Δ*FvAtg4-8* strains, mycelia of each strain were grown in liquid GYAM medium (0.24 M glucose, 0.05% yeast extract, 8 mM L-asparagine, 5 mM malic acid, 1.7 mM NaCl, 4.4 mM K_2_HPO_4_, 2 mM MgSO_4_, and 8.8 mM CaCl_2_, pH 3.0) for 7 d [34]. The intracellular alanine was extracted and quantified with an alanine assay kit (Sigma, MO, USA).

### FB1 production assays

To determine FB1 production from cracked maize, batches of healthy maize kernels (25 g) were sterilized and inoculated individually with each strain of *F. verticillioides* at room temperature for three weeks. FB1 was extracted and assayed as previously described [35]. The amount of FB1 (per g maize kernel tissue) was determined with a Waters 1525 High Performance Liquid Chromatography (HPLC) system and liquid chromatography/mass spectrometry (LC-MS) using a previously described method [27]. The amount of ergosterol was evaluated by the method of Yin et al [36]. and used to normalize the production of FB1. To detect FB1 production in GYAM, five agar plugs of the *F. verticillioides* strain were grown in GYAM at room temperature with shaking (200 rpm) for 7 d. Cultures were filtered through 0.2 μM Nalgene filters and analyzed as described previously by HPLC and LC-MS [34].

### RNA extraction and quantitative real-time PCR (qRT-PCR)

Harvested mycelia were lyophilized and ground to powder in liquid nitrogen for total RNA extraction using TaKaRa RNAiso Reagent. Ten μg of each total RNA sample was then used for reverse transcription using a RevertAid H Minus First Strand cDNA Synthesis Kit (Fermentas Life Sciences, Burlington, Canada). qRT-PCR was performed to detect the expression levels of each gene such as *FvBIK1* to *FvBIK6* using the primers listed in Table S1. Expression values are presented as the values relative to the expression in 7600 and the deletion mutants (Δ*FvAtg4*, Δ*FvAtg8*, and Δ*FvAtg4-8*). Three independent biological replicates using three different RNA preparations were analyzed to calculate the means and standard deviations using the 2^−ΔΔCT^ Method [37].

### Yeast two-hybrid assays

The coding sequences of *FvATG4* and *FvATG8* were amplified from cDNA of *F. verticillioides* 7600 and further inserted into the vector pGBKT7 and the GAL4 activation domain vector pGADT7. Both plasmids were cotransformed into *S. cerevisiae* strain AH109 using the LiAc/SS-DNA/PEG transformation protocol [38]. Transformants were grown at 30°C for 3 d on synthetic medium lacking Leu and Trp and then transferred to medium depleted of His, Leu, and Trp and containing 5 mM 3-aminotriazole (3-AT) to assess binding activity.

## Results

### Deletion and complementation of FvATG4 and FvATG8 in *F.* *verticillioides*

We used the sequences of the *S. cerevisiae* Atg4 and Atg8 proteins as queries in BLASTp searches and identified their orthologs, FVEG_05096 (named FvATG4) and FVEG_11401 (named FvATG8), in the *F. verticillioides* genome. The amino acid sequences of the predicted FvAtg4 and FvAtg8 proteins shared 59.5% and 79.3% identity with the sequences of *S. cerevisiae* Atg4 and Atg8, respectively (Figure S1a, b). The phylogenetic analysis showed that the Atg4 and Atg8 orthologs clustered into different groups (Figure S1c). FvAtg4 and FvAtg8 deletion mutants were generated using a homologous recombination strategy (Figure S2a). The deletion mutants were further confirmed by Southern blot analysis (Figure S2b). To confirm that the phenotypic changes observed in Δ*FvAtg4* and Δ*FvAtg8* were caused by the deletion of *FvATG4* and *FvATG8*, complementation was performed by reintroduction of full-length *FvATG4* and *FvATG8* into their deletion mutants (Δ*FvAtg4* and Δ*FvAtg8*, respectively).

### Subcellular localization of FvAtg4 and FvAtg8 during conidial and vegetative growth in *F.* *verticillioides*

**Figure 1.**
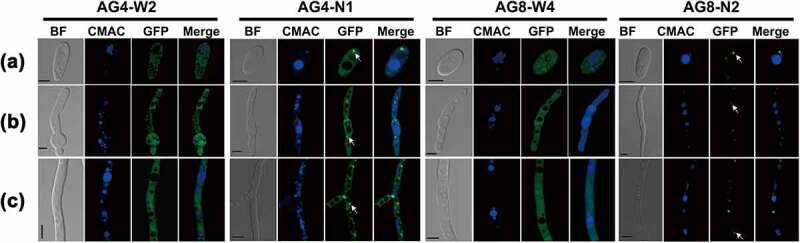
Expression and subcellular localization of FvAtg4 and FvAtg8 in *F. verticillioides* conidia (a), germ tubes (b), and hyphae (c). *F. verticillioides* strains expressing the FvAtg4-GFP or GFP-FvAtg8 fusion protein constructs and stained with CMAC were examined by epifluorescence microscopy. AG4-N1 carried the FvAtg4-GFP fusion protein in the Δ*FvAtg4* background, and AG8-N2 carried the GFP- FvAtg8 fusion protein in the Δ*FvAtg8* background. as a control, GFP was expressed from constructs under the control of the native promoters of FvATG4 and FvATG8. the GFP fluorescence in the resulting transformants, AG4-W2 and AG8-W4, was dispersed throughout the cytoplasm. White arrows indicate the punctate structures. BF=Bright Field. Scale bars = 5 μm.

The FvAtg4-GFP signal was detected during conidiation, conidial germination, and vegetative growth and was mainly localized in punctate structures (Figure 1, AG4-N1). The GFP-FvAtg8 fusion protein was also expressed during asexual development and exclusively localized in punctate structures in conidia, germinated conidia, and vegetative hyphae (Figure 1, AG8-N2). As a control, the expression of GFP driven by the FvAtg4 or FvAtg8 promoter was detected in conidia, conidial germination, and hyphal growth, and GFP fluorescence was found to be dispersed throughout the cytoplasm (Figure 1, AG4-W2 and AG8-W4). Taken together, these results indicate that FvAtg4 and FvAtg8 are constantly expressed during fungal development and seem to be mainly localized to punctate structures.

### Deletion of FvATG4 and FvATG8 inhibits the autophagic pathway in *F.* *verticillioides*

In *S. cerevisiae*, Atg4 and Atg8 are essential for the formation of autophagic bodies. To determine whether FvAtg4 and FvAtg8 function similarly to the *S. cerevisiae* orthologs, expression vectors carrying full-length cDNA sequences of *FvATG4* and *FvATG8* were individually transformed into the yeast mutants Δ*Atg4* and Δ*Atg8*, respectively. RT-PCR with the primer pairs A13 + A14 and A15 + A16 amplified the expected 445 bp and 308 bp sequences in the Δ*Atg4* + FvATG4-pYES2 and Δ*Atg8* + FvAtg8*-pYES2 S. cerevisiae* strains, respectively, but did not amplify anything from the strains BY4741 + pYES2, Δ*Atg4* + pYES2, and Δ*Atg8* + pYES2 (Figure S3a), indicating the introduction of *FvATG4* or *FvATG8* into the corresponding *S. cerevisae* mutants. The pYES2 vector was introduced into *S. cerevisiae* BY4741 as a negative control. FvAtg4 and FvAtg8 complemented the *S. cerevisiae* Δ*Atg4* and Δ*Atg8* strain defects in the accumulation of autophagic bodies when they were cultured in SD-N liquid medium in the presence of 1 mM phenylmethanesulfonyl fluoride (PMSF) (Figure S3b).

**Figure 2.**
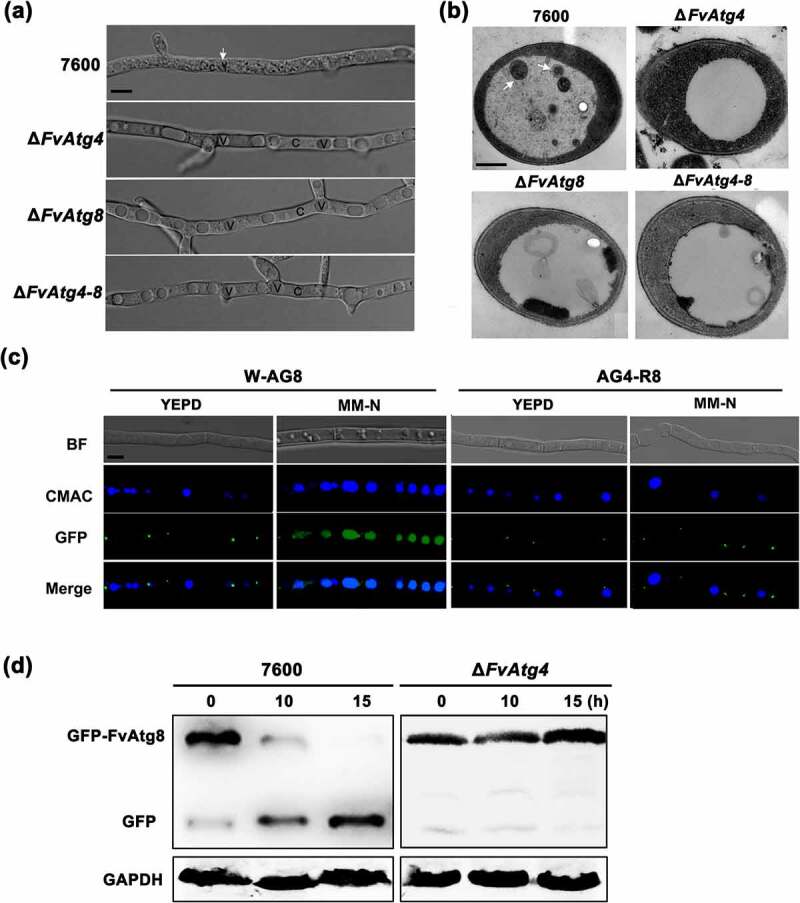
Assay for autophagy defects in the Δ*FvAtg4* and Δ*FvAtg8* mutants. (a) the vacuoles were filled with autophagosomes in the wild-type 7600 strain, while no autophagic bodies could be observed in the vacuoles of the Δ*FvAtg4*, Δ*FvAtg8* and Δ*FvAtg4-8* deletion mutants after growth in MM-N medium supplemented with 1 mM PMSF for 8 h. White arrows indicate the autophagic bodies. “V” is vacuole and “C” is cytoplasm. Scale bar = 5 μm. (b) Organelles and autophagic bodies in the wild-type 7600, Δ*FvAtg4*, Δ*FvAtg8*, and Δ*FvAtg4-8* mutant strains were observed under starvation conditions. White arrows indicate the autophagic bodies. Scale bar = 0.5 μm. (c) W-AG8 and AG4-R8 carried the GFP- FvAtg8 fusion protein into wild type 7600 or the Δ*FvAtg4* background, respectively. Strains were cultured in liquid YEPD and transferred into liquid MM-N medium. Mycelia stained with CMAC were observed using epifluorescence microscopy. BF=Bright Field. Scale bar = 10 μM. (d) GFP-FvAtg8 degradation was verified using an immunoblot assay with anti-GFP antibody in the wild-type 7600 and Δ*FvAtg4* strains. Mycelia were harvested at each time point indicated during nitrogen starvation. GAPDH was used as a reference protein.

Autophagy in Δ*FvAtg4*, Δ*FvAtg8*, Δ*FvAtg4-8*, and wild-type *F. verticillioides* 7600 was examined by observing the nutrient-starved hyphae under a differential interference microscope. No autophagic bodies were observed in the vacuoles of the Δ*FvAtg4*, Δ*FvAtg8*, and Δ*FvAtg4-8* when induced on minimal medium lacking an N source (MM-N) and supplemented with 1 mM PMSF for 8 h (Figure 2a). However, autophagic bodies could be observed in the wild-type strain 7600. To further confirm the blockade of autophagy in Δ*FvAtg4*, Δ*FvAtg8*, and Δ*FvAtg4-8*, we examined the autophagic bodies in the vacuoles of each strain by transmission electron microscopy. Compared to the wild-type 7600, the Δ*FvAtg4*, Δ*FvAtg8*, and Δ*FvAtg4-8* showed defects in the accumulation of autophagic bodies in the lumen of the vacuoles (Figure 2b).

The process of autophagic flux could be detected by monitoring the breakdown of GFP-Atg8 in the fungi. The autophagosome is cleaved by vacuolar hydrolases when the autophagic flux is normal. GFP cleaved from GFP-Atg8 is then resistant to hydrolases and accumulates in the vacuole [9]. In *F. verticillioides*, we observed that the fluorescent GFP-FvAtg8 signal was localized to punctate structures under non-inducing conditions (YEPD medium) in both the wild-type 7600 and the Δ*FvAtg4* strains, but no GFP signal was detected in the vacuole lumens (Figure 2(c)). When cultured under nitrogen starvation conditions (MM-N medium) for 4 h, the GFP signal was distributed throughout the CMAC-stained vacuoles in the wild-type strain, while no GFP was detected in the vacuoles of Δ*FvAtg4*. Next, we performed a GFP-FvAtg8 proteolysis assay to monitor the process of autophagy in the Δ*FvAtg4* mutant. The protein levels of full-length GFP-FvAtg8 were significantly decreased in the wild-type 7600 strain but not in Δ*FvAtg4* after the strains were shifted from YEPD to MM-N (Figure 2(d)), suggesting that FvAtg4 is important for the induction of autophagy in *F. verticillioides*.

We also examined the subcellular localization of FvAtg4-GFP in the wild-type and Δ*FvAtg8* strains to test whether FvAtg8 associates with the subcellular localization of FvAtg4. There was no obvious difference in the subcellular localization of FvAtg4-GFP in the wild-type 7600 or the Δ*FvAtg8* strains under inducing or non-inducing conditions (Figure S4).

### FvAtg4 interacts with FvAtg8 in *F.* *verticillioides*

**Figure 3.**
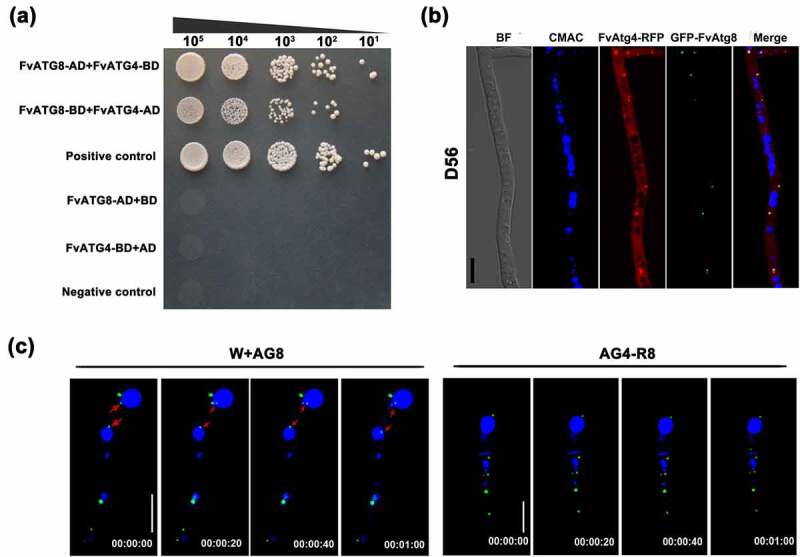
The FvAtg4 protein interacts with the FvAtg8 protein in *F. verticillioides*. (a) Serial dilutions of yeast cells (cells/ml) transformed with the bait and prey constructs indicated on the left were assayed for growth on SD-Leu-Trp-His plates. a pair of plasmids, pGBKT7–53 and pGADT7, was used as the positive control. a second pair of plasmids, pGBKT7-Lam and pGADT7, was used as the negative control. (b) Colocalization of FvAtg4-red fluorescent protein (FvAtg4-RFP) with green fluorescent protein-FvAtg8 (GFP-FvAtg8) in *F. verticillioides*. the pair of plasmids FvAtg4-RFP and GFP-FvAtg8 were cotransformed into the wild-type 7600 strain, and the resulting transformant was examined for GFP and RFP signals. (c) Effects of *FvATG4* deletion on the cellular movements of GFP-FvAtg8 during conidial germination. Germinated conidia of the wild-type 7600 strain and the *FvATG4* deletion mutant expressing the GFP-FvAtg8 fusion construct were stained with CMAC and examined by confocal epifluorescence microscopy. Red arrows indicate the relative position of GFP-FvAtg8 at each time point. BF=Bright Field. Scale bars = 5 μM.

In *S. cerevisiae*, Atg4 has been shown to directly interact with Atg8 [21,39]. To examine whether FvAtg4 interacts with FvAtg8 in *F. verticillioides*, we performed a yeast two-hybrid assay and found that FvAtg4 could interact with FvAtg8 in *F. verticillioides* (Figure 3(a)). To further analyze the relationship between FvAtg4 and FvAtg8, colocalization of FvAtg4 and FvAtg8 was observed under confocal epifluorescence microscopy. A pair of plasmids, FvAtg4-RFP and GFP-FvAtg8, were cotransformed into wild-type 7600. The FvAtg4-RFP and GFP-FvAtg8 fusion proteins were colocalized in punctate structures in the cotransformed strain D56 (Figure 3(b)), suggesting a direct interaction between FvAtg4 and FvAtg8, as in the budding yeast.

In addition, time-lapse images of the wild-type strain revealed that the GFP-FvAtg8 fusion protein translocated into the vacuoles during conidial germination, but GFP-FvAtg8 was retained in punctate structures and failed to translocate into vacuoles upon loss of FvAtg4 function (Figure 3(c)). This result suggested that FvAtg4 mediates the translocation of FvAtg8 into vacuoles.

### FvAtg4 and FvAtg8 are important regulators of lipid turnover

**Figure 4.**
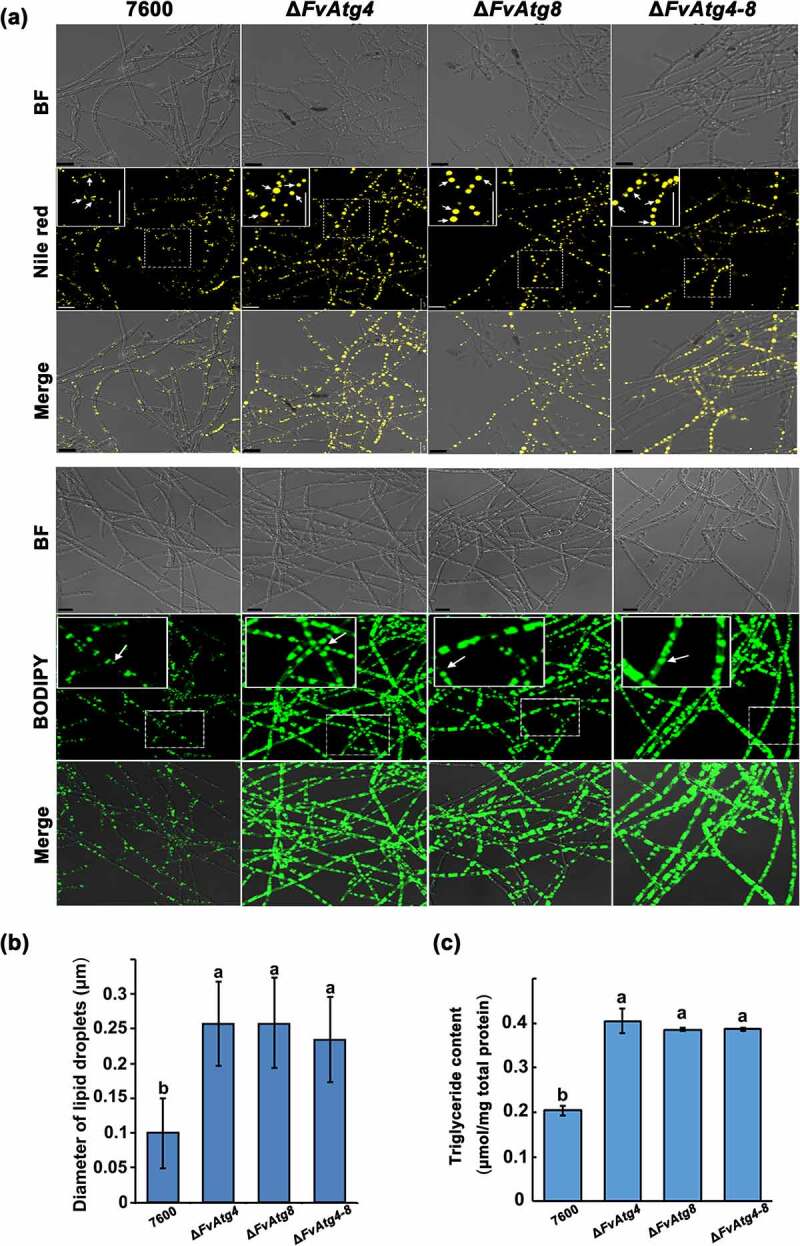
Involvement of FvAtg4 and FvAtg8 in lipid turnover in *F. verticillioides*. (a) Mycelia of the wild-type 7600 strain, Δ*FvAtg4*, Δ*FvAtg8* and Δ*FvAtg4-8* were grown in liquid MM medium and stained with Nile red or BODIPY to observe the lipid droplets in the hyphae. Arrows indicate lipid droplets in the enlarged views. BF=Bright Field. Scale bars = 10 μm. (b) Lipid droplet sizes of the wild-type 7600 strain, Δ*FvAtg4*, Δ*FvAtg8* and Δ*FvAtg4-8* were measured. (c) the amount of triglycerides in each strain was determined using a commercially available enzymatic assay kit. Error bars in each column indicate the standard error of three independent experiments. Different letters indicate a significant difference (*P* < .05).

The intracellular storage and utilization of lipids are critical for virulence in pathogenic fungi. Cellular lipids are stored as triglycerides (TGs) in lipid droplets (LDs) that play key roles in lipid metabolism and in energy storage and balance in eukaryotic cells [40]. Indeed, many of the enzymes that synthesize phospholipids (PLs), triacylglycerols (TGs), and their intermediates, as well as lipases and lipolytic regulators, are localized on LD surfaces. In this study, the accumulation of lipid droplets was compared by staining mycelia of each strain with Nile red- or BODIPY and observed using a confocal laser scanning microscope. A significant increase in lipid droplet size was observed in Δ*FvAtg4* and Δ*FvAtg8* compared to wild-type (Figure 4(a,b)). We also found a significant increase in TG production in Δ*FvAtg4*, Δ*FvAtg8*, and Δ*FvAtg4-8* (Figure 4(c)), indicating that FvAtg4 and FvAtg8 play essential roles in the regulation of TG accumulation.

### FvAtg4 and FvAtg8 are involved in hyphal growth and pigmentation in *F.* *verticillioides*

**Figure 5.**
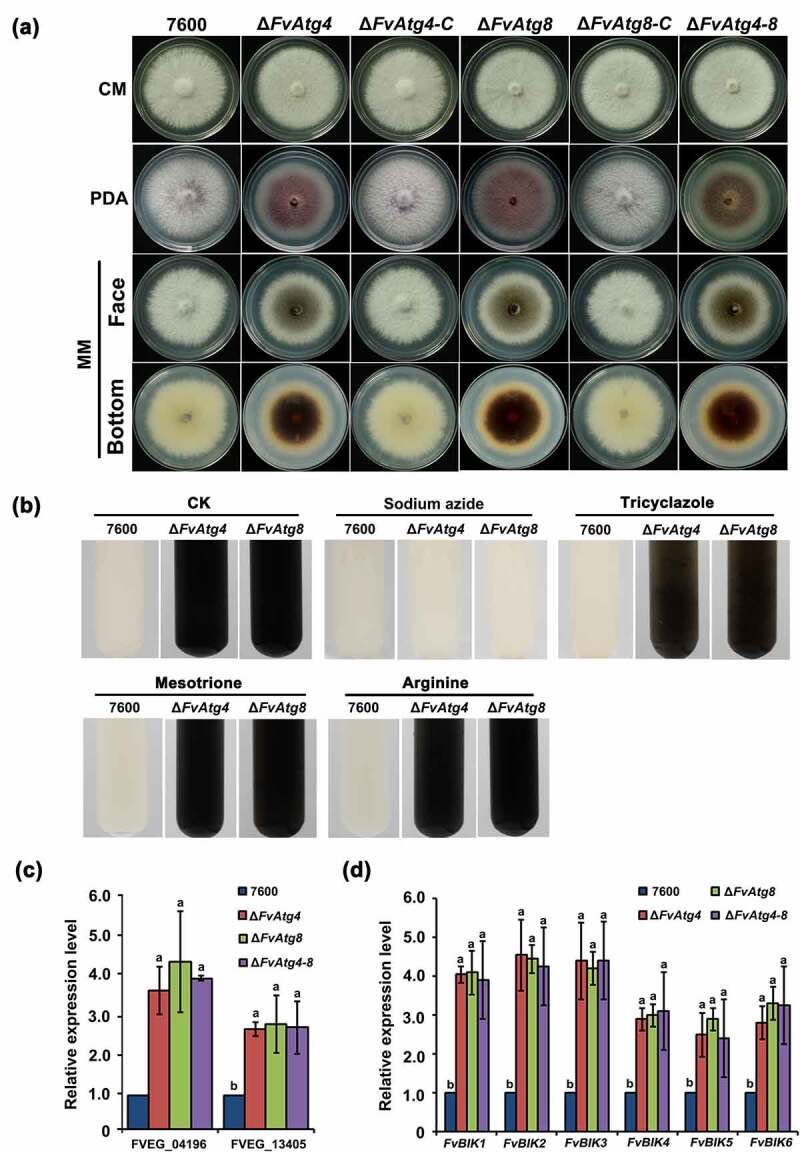
Effects of *FvATG4* and *FvATG8* deletion on mycelial growth and pigmentation in *F. verticillioides*. (a) *F. verticillioides* strains were grown on CM, PDA and MM media at 25°C for 4 d. (b) Effect of four inhibitors on the melanin biosynthesis of the wild-type 7600 strain, ∆fvatg4 and ∆fvatg8. Each strain was incubated in liquid MM and treated with 0.2 mM sodium azide (inhibits laccase in the Raper-Mason pathway), 50 μg/mL tricyclazole (inhibits the dehydrogenation reaction in the DHN pathway), 50 μg/mL mesotrione (inhibits the HGA pathway), or 0.5 mM arginine (inhibits tyrosinase inhibitors in the Raper-Mason pathway). (c) Relative expression of two laccase genes, FVEG_04196 and FVEG_13405, in wild-type *F. verticillioides* 7600, ∆*FvAtg4*, ∆*FvAtg8* and the ∆*FvAtg4-Atg8* double mutant. the expression levels of the laccase genes in ∆*FvAtg4*, ∆*FvAtg8* and ∆*FvAtg4-Atg8* are the relative amounts of mRNA in the wild-type progenitor strain. (d) Relative expression of the *FvBIK* genes in the wild-type 7600 strain and the ∆fvatg4, ∆fvatg8 and ∆fvatg4–8 deletion mutants. Error bars in each column indicate the standard error from three separate experiments. Different letters indicate a significant difference (*P* < .05).

Hyphal growth and colony morphology remained unchanged in the mutants (Δ*FvAtg4*, Δ*FvAtg8*, and Δ*FvAtg4-8*) as compared to the wild-type 7600 strain when cultured on CM. However, all the mutants showed significantly decreased hyphal growth compared with the wild type on PDA and MM (Figure 5(a)). Microscopic examinations showed that the mutants failed to form normal aerial hyphae on PDA and MM (Figure S5). In contrast to the wild-type, Δ*FvAtg4*, Δ*FvAtg8*, and Δ*FvAtg4-8* mutants produced more black pigment on MM, suggesting that the biosynthesis of a melanin pigment might be increased (Figure 5(a,b)). Melanin is a black pigment that occurs widely in fungal species. To confirm the presence of melanin in the mutants, we extracted and purified the black pigment from each mutant and compared them to synthetic melanin using Fourier transform-infrared (FT-IR) spectroscopy. Similar spectra and functional groups were detected between the extracted black pigments and synthetic melanin, indicating that the extracted black pigments are melanin pigments (Figure S6 and Table S2).

To confirm that FvAtg4 and FvAtg8 are involved in melanin production, we treated Δ*FvAtg4* and Δ*FvAtg8* with different melanin synthesis inhibitors and found that only sodium azide (an inhibitor of laccase in the Raper-Mason pathway) dramatically inhibited melanin synthesis in the mutants (Figure 5(b)). The expression levels of two putative laccase genes, FVEG_04196 and FVEG_13405, were found to be significantly upregulated in the *F. verticillioides* Δ*FvAtg4*, Δ*FvAtg8*, and Δ*FvAtg4-8* (Figure 5(c)). These results suggested that FvAtg4 and FvAtg8 negatively regulate melanin biosynthesis via the downregulation of laccase genes in *F. verticillioides*.

We also found that the mutants (Δ*FvAtg4*, Δ*FvAtg8*, and Δ*FvAtg4-8*) displayed dramatically increased red pigments when cultured on PDA plates compared to the wild-type and complemented strains (Figure 5(a) and Figure S7a). HPLC-MS analysis showed a significant increase in bikaverin production in the three deletion mutants (Figure S7b). It has been predicted that *FvBIK* gene cluster consisting of six genes, *FvBIK1* to *FvBIK6* (FVEG_03379 to FVEG_03384), they are important for the biosynthesis of the red pigment bikaverin [41]. We checked the expression of the *FvBIK* genes that was significantly increased in the deletion mutants in comparison with the wild-type (Figure 5(d)). To further confirm the finding that the increased red pigments in the deletion mutants were due to the upregulation of *FvBIK* gene transcripts, we generated double deletion mutants for *FvBIK1* and *FvBIK3* and measured bikaverin production. All the resulting double deletion mutants (Ag4-bk3, Ag8-bk3, Ag4-bk1, and Ag8-bk1) showed significantly decreased production of red pigments on PDB compared to the Δ*FvAtg4* and Δ*FvAtg8* strains (Figure S7a), suggesting that FvAtg4 and FvAtg8 are involved in bikaverin biosynthesis regulation in *F. verticillioides*.

### FvAtg4 and FvAtg8 are required for pathogenicity in *F.* *verticillioides*

**Figure 6.**
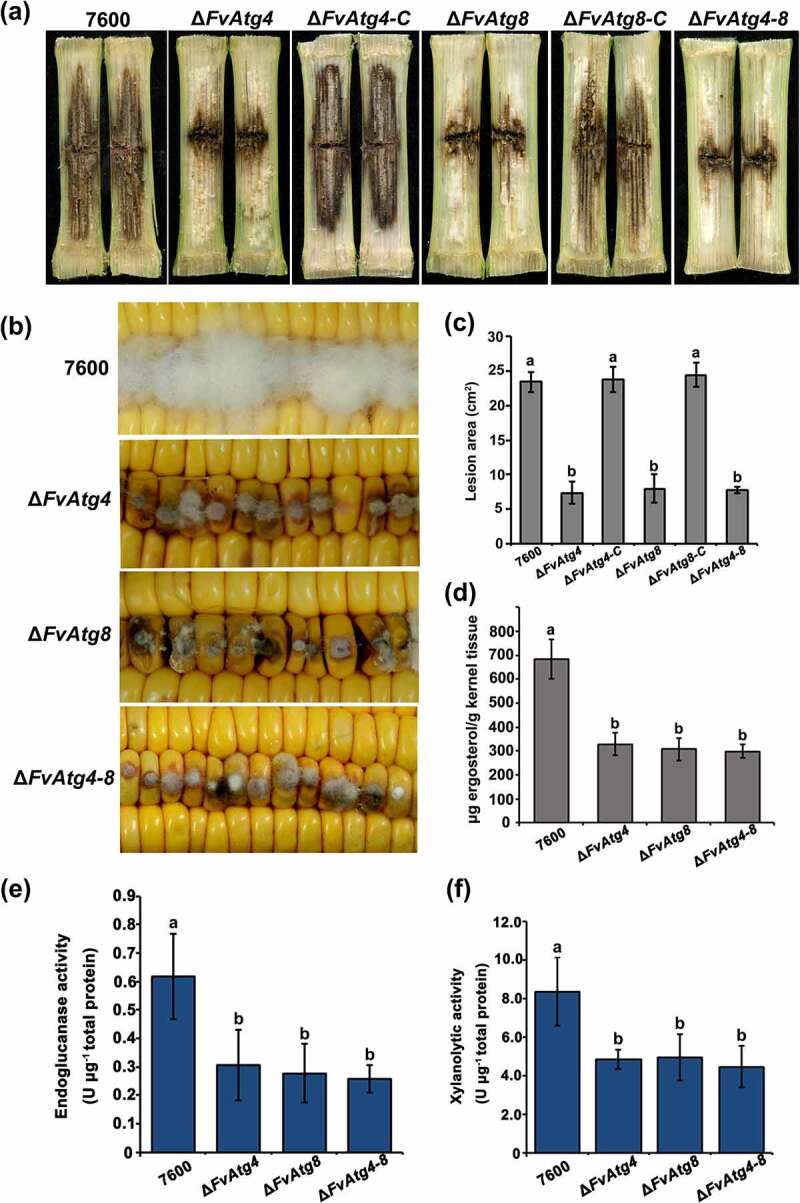
FvAtg4 and FvAtg8 are required for full virulence in *F. verticillioides*. (a) Disease symptoms of the wild-type 7600 strain, Δ*FvAtg4*, Δ*FvAtg8*, Δ*FvAtg4-8*, Δ*FvAtg4-C* and Δ*FvAtg8-C* on maize stalks 15 d after inoculation and (b) on wounded maize kernels 7 d after inoculation. (c) Lesion areas of longitudinally dissected maize stalks infected with *F. verticillioides* measured after 15 d. (d) Ergosterol production in each strain on maize kernels. Endoglucanase activity (e) and xylanolytic enzyme activity (f) in the wild-type 7600 strain, Δ*FvAtg4*, Δ*FvAtg8*, and Δ*FvAtg4-8*. Error bars denote the standard error of three separate experiments. Different letters indicate a significant difference (*P* < .05).

Maize stalk infection assays showed that at 15 days post inoculation (dpi), the maize stalk rot symptoms caused by Δ*FvAtg4*, Δ*FvAtg8*, and Δ*FvAtg4-8* were significantly reduced compared with the wild-type and complemented strains (Figure 6(a,c)). In the kernel assay, the Δ*FvAtg4*, Δ*FvAtg8*, and Δ*FvAtg4-8* mutant strains formed fewer aerial hyphae at 7 dpi than the wild-type strain (Figure 6(b)). Furthermore, we found that the deletion mutants (Δ*FvAtg4*, Δ*FvAtg8*, and Δ*FvAtg4-8*) were impaired in fungal growth when grown in maize kernel medium and maize stem medium (Figure S8). We measured the amount of ergosterol in the *F. verticillioides* strains and found a significant reduction in the Δ*FvAtg4*, Δ*FvAtg8*, and Δ*FvAtg4-8* mutant strains compared with the wild-type 7600 strain (Figure 6(d)). To further explore the roles of FvAtg4 and FvAtg8 in pathogenicity, we assayed the Cell Wall Degrading Enzyme (CWDE) activities and found that the mutants exhibited a significant reduction in endoglucanase and xylanolytic activities (Figure 6(e,f)). Taken together, these findings suggest that FvAtg4 and FvAtg8 may regulate fungal virulence by affecting fungal growth and certain CWDE activities.

### FvAtg4 and FvAtg8 are required for sexual but not asexual growth in *F.* *verticillioides*

The *FvATG4* and *FvATG8* deletion mutants showed no detectable changes in the morphologies of either asexual micro- or macroconidia (data not shown), the production of micro- and macroconidia, or in conidial germination (Figure S9a,b). To explore the roles of FvAtg4 and FvAtg8 in sexual development, each strain was crossed with the opposite mating type, strain 7598. After 21 d of incubation, the wild-type 7600 strain displayed normal perithecia production. However, the three deletion mutants failed to produce perithecia (Figure S9c), suggesting that FvAtg4 and FvAtg8 play indispensable roles in the sexual development of *F. verticillioides.*

### FvAtg4 and FvAtg8 are required for FB1 biosynthesis in *F.* *verticillioides*

**Figure 7.**
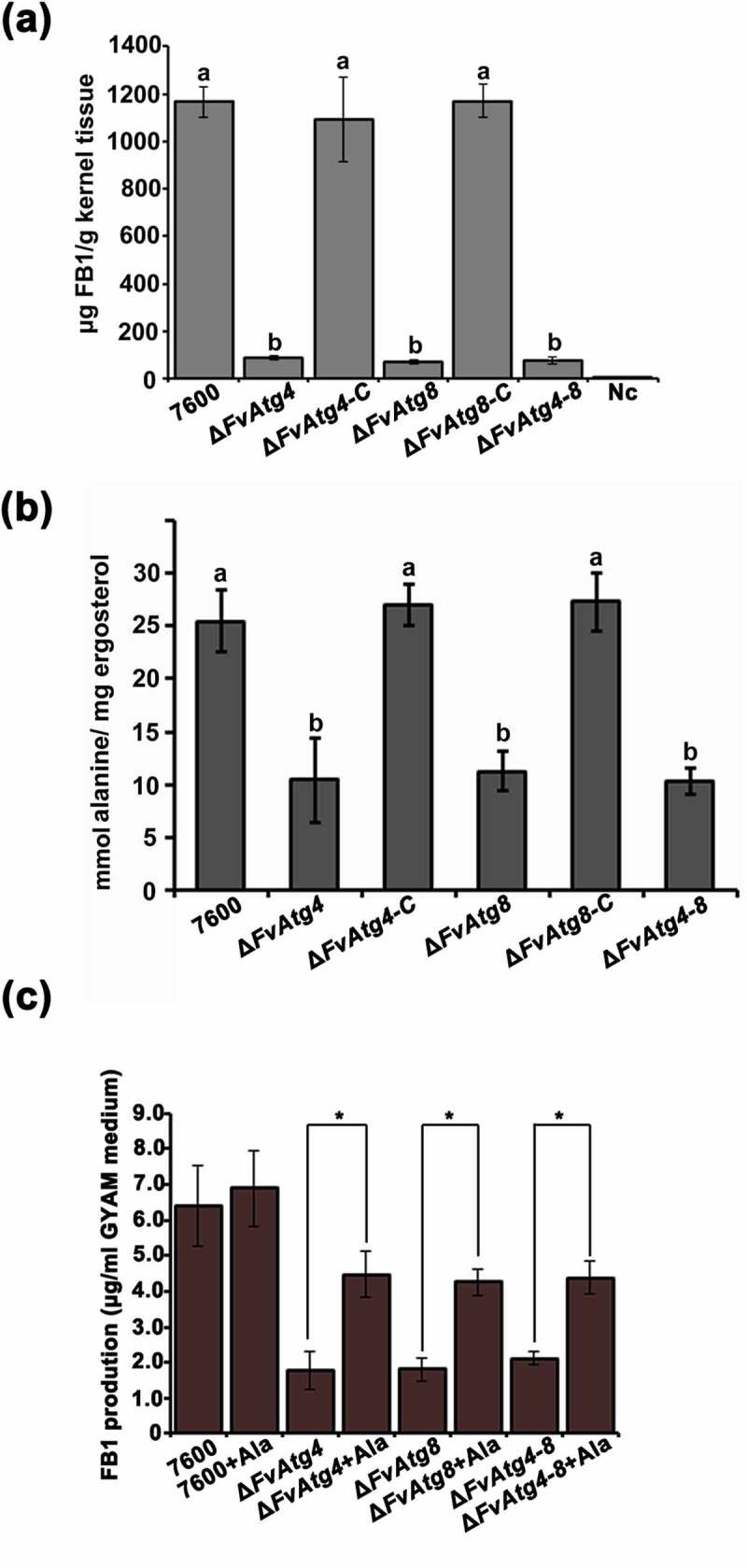
Effects of the *FvATG4* and *FvATG8* deletions on FB1 biosynthesis in *F. verticillioides*. (a) The amounts of FB1 (per g maize kernel tissue) produced in infected maize kernels by the *F. verticillioides* strains 21 d after inoculation. (b) the amount of alanine (per mg fungal ergosterol) produced by each strain after growth in GYAM medium at 25°C for 7 d. (c) Effect of alanine on the FB1 production of the wild-type and the mutants incubated in GYAM medium. Error bars in each column indicate the standard error of three independent experiments. Different letters indicate a significant difference (*P* < .05).

To characterize the effects of the *FvATG4* and *FvATG8* deletions on FB1 biosynthesis, we analyzed FB1 by LC-MS in the mutant strains grown on cracked maize kernels and in GYAM medium. Twenty-one days after maize kernel inoculation, FB1 production in the Δ*FvAtg4* and Δ*FvAtg8* mutants was markedly decreased (Figure 7(a)) as compared with the wild type 7600. Similar defects in FB1 production were found in the mutants after 7 d of incubation in GYAM medium. The *FUM* gene cluster is responsible for FB1 biosynthesis and regulation in *F. verticillioides* [42]. We compared the transcript levels of the *FUM* genes between the mutant strains and the wild-type 7600 strain by qRT-PCR after incubation in liquid GYAM medium or on maize kernels. There were no obvious changes in the expression the *FUM* genes in the mutant strains compared to the wild-type 7600 strain (Figure S10a,b). To further study the effects of *FvATG4* or *FvATG8* deletion on FB1 biosynthesis, we measured the amount of alanine, a major precursor in the biosynthesis of FB1, in each *F. verticillioides* strain. We found a significant reduction in the alanine levels of the Δ*FvAtg4*, Δ*FvAtg8* and Δ*FvAtg4-8* strains when cultured in GYAM medium (Figure 7(b)). We supplemented the GYAM cultures with exogenous alanine and found that FB1 production in the mutants was significantly increased (Figure 7(c)), suggesting that alanine could partially rescue defective FB1 biosynthesis in the mutants.

## Discussion

In eukaryotic cells, autophagy is required for the recycling of cellular components and serves as a survival strategy during starvation [43]. To date, autophagy has been shown to play important roles in cellular differentiation and virulence in several pathogenic and model fungi, including *M. oryzae*, *F. graminearum*, and *P. anserine* [9,10,14]. In this study, we identified the *FvATG4* and *FvATG8* genes and characterized the roles of the FvAtg4 and FvAtg8 proteins in vegetative growth, fungal virulence and FB1 biosynthesis in the important maize pathogen *F. verticillioides.*

In *S. cerevisiae*, more than 30 autophagy-related proteins have been identified [44–46]. Deletion of *ATG4* or *ATG8* resulted in defects in autophagosome formation in *S. cerevisiae* [46]. In the current study, *FvATG4* and *FvATG8* rescued the defects in autophagosome formation of *S. cerevisiae ATG4* and *ATG8* null mutants, indicating that the Atg4 and Atg8 proteins have conserved functions in both yeast and *F. verticillioides*. Moreover, we found that deletion of *FvATG4* or *FvATG8* led to similar defects in the formation of autophagosomes in *F. verticillioides* as in *S. cerevisiae* and other filamentous fungi [10]. All these results provide evidence that Atg4 and Atg8 play essential roles in autophagosome formation, which is conserved from yeast to filamentous fungi.

In *S. cerevisiae*, Δ*Atg4* and Δ*Atg8* mutants are defective in stress-induced differentiation and development [47]. *ATG4* and *ATG8* mutants also showed defects in vegetative growth, conidiation, and sexual development in filamentous fungi [9,10]. In this study, similar to other filamentous fungi, deletion of *F. verticillioides FvATG4* or *FvATG8* resulted in reduced vegetative growth in nutrient-limited medium and blocked perithecia formation, indicating conserved roles of Atg4 and Atg8 in cellular differentiation in yeast and filamentous fungi. However, we found that FvAtg4 and FvAtg8 were not involved in conidiation or conidial germination in *F. verticillioides*, which is different from budding yeast and other filamentous fungi.

Autophagy plays distinct roles in the pathogenesis of diverse pathogenic fungi. In *M. oryzae*, Atg4 and Atg8 mutants were attenuated in appressorium-mediated pathogenicity [25]. In *F. graminearum*, FgAtg8 was shown to be necessary for non assimilating fungal structures and plant colonization [14]. However, the autophagy-related protein AfAtg1 in *A. fumigatus* was not involved in fungal pathogenesis [24]. Similarly, the deletion of *CaATG9* in *Candida albicans* resulted in defects in autophagy, but the Δ*CaAtg9* mutant was fully virulent compared with the wild-type strain [48]. In this study, the reduced pathogenicity of Δ*FvAtg4* and Δ*FvAtg8* on maize stalks and kernels may result from multiple phenotypic defects, including reduced hyphal growth and decreased lipid turnover, in the mutants. Furthermore, the *F. verticillioides* deletion mutants also had significantly reduced endoglucanase and xylanolytic activities, which may partially explain their reduced virulence in maize. The development of pathogenesis in filamentous fungi requires intracellular storage and utilization of lipids [49], and autophagy was found to be an important regulator of lipid metabolism in mammalian cells [50]. Moreover, autophagy-dependent lipid breakdown is considered to be required to generate glycerol needed to keep turgor pressure in aerial mycelia and mycelia growing over hydrophobic surfaces [14]. Here, we also found that autophagy is important for lipid turnover in *F. verticillioides*, which may partially explain the defects of pathogenicity in the mutants.

Melanin, which is widely found in pathogenic fungi, is crucial in fungal pathogenesis and acts as a protective mechanism against diverse environmental insults [51–53]. The deletion of *FvATG4* or *FvATG8* resulted in increased melanin production. We found a significant upregulation of two predicted laccase genes in the deletion mutants, which might result in the increased production of melanin in the mutants. This result indicates that autophagy may control melanin biosynthesis by negatively regulating melanin biosynthesis-related genes. In addition to increased levels of melanin, we also found increased production of red pigment (bikaverin) in the deletion mutants, which might result from the overexpression of *FvBIK* genes in the deletion mutants. These findings indicate that autophagy plays a negative role in the biosynthesis of pigments, including melanin and bikaverin.

In *S. cerevisiae*, Atg4 interacts directly with Atg8 by cleaving its carboxyl-terminal end, and this truncated Atg8 is indispensable for the conjugation of Atg8-PE [21,39]. Similar to *S. cerevisiae*, we also found a direct interaction between FvAtg4 and FvAtg8. Colocalization observations also showed an interaction between FvAtg4 and FvAtg8 in punctate structures. Furthermore, the GFP-FvAtg8 fusion protein failed to move into the vacuoles in the Δ*FvAtg4* mutants during conidial germination, indicating an indispensable role for FvAtg4 in the translocation of FvAtg8, while the subcellular localization of FvAtg4 in *F. verticillioides* did not change. These data indicated that the proper subcellular localization of FvAtg4 and FvAtg8 is important for their biological functions.

In summary, identification and characterization of FvAtg4 and FvAtg8 in *F. verticillioides* revealed crucial and conserved roles of these two autophagy-related proteins in fungal growth and plant infection among plant pathogenic fungi. Moreover, this study provided the evidence of autophagy regulating the production of fungal secondary metabolites. Previous studies have showed that *F. graminearum* autophagy related proteins negatively regulated red pigment and deoxynivalenol (DON) production [14]. Unlike its counterparts in *F. graminearum*, in this study, we found that FvAtg4 and FvAtg8 affected the levels of intracellular precursor alanine production, thus displayed positive regulatory roles on FB1 biosynthesis. The expression of FB1 biosynthetic genes were not affected in the *FvATG4* and *FvATG8* mutants. Thus, we hypothesize that autophagy may control FB1 biosynthesis by regulating alanine recycling. The production of melanin-like pigment and bikaverin was found to be negatively regulated by FvAtg4 and FvAtg8 by affecting the expression of melanin biosynthesis-related genes and *FvBIK* genes. In this study, we observed that decreased levels of FB1 were produced by Δ*FvAtg4* and Δ*FvAtg8*. The expression levels of the *FUM* genes were not significantly affected, but the levels of intracellular alanine decreased in the Δ*FvAtg4* and Δ*FvAtg8* mutants. Taken together, we show that autophagy plays a significant role in fungal growth, secondary metabolism and plant infection in *F. verticillioides*, which advances our understanding of autophagy in plant pathogenic fungi.

## Supplementary Material

Supplemental Material

## Data Availability

All relevant data are within the manuscript and its Supporting Information files.
