# Supplementary material for: The autophagy-related proteins FvAtg4 and FvAtg8 are involved in virulence and fumonisin biosynthesis in Fusarium verticillioides
Source: Virulence. 2022 Apr 20;13(1):764–80. doi: 10.1080/21505594.2022.2066611 (PMC9067522; doi:10.1080/21505594.2022.2066611)
Supplement: Supplemental Material [file KVIR_A_2066611_SM0326.docx]

**Autophagy-related FvAtg4 and FvAtg8 are involved in lipid turnover, virulence, and fumonisin biosynthesis in *Fusarium verticillioides***

YUJIE WANG^1#^, XIN LIU^2, 3#^, YUJIAO XU^1#^, YIYING GU^1^, XINYUE ZHANG^1^, MENGXUAN ZHANG^1^, WEN WEN^1^, YIN-WON LEE^4^, JIANRONG SHI^2, 3^, SHERIF RAMZY MOHAMED^5^, AMIRA A. GODA^5^, HUIJUN WU^1^, QIN GU^1*^, AND XUEWEN GAO^1^

**^1^** Department of Plant Pathology, College of Plant Protection, Nanjing Agricultural University/Key Laboratory of Monitoring and Management of Crop Diseases and Pest Insects, Ministry of Education, Nanjing 210014, Jiangsu, China.

^2^ Jiangsu Key Laboratory for Food Quality and Safety-State Key Laboratory Cultivation Base, Ministry of Science and Technology/Key Laboratory for Control Technology and Standard for Agro-product Safety and Quality, Ministry of Agriculture and Rural Affairs/Key Laboratory for Agro-product Safety Risk Evaluation (Nanjing), Ministry of Agriculture and Rural Affairs/Collaborative Innovation Center for Modern Grain Circulation and Safety/Institute of Food Safety and Nutrition, Jiangsu Academy of Agricultural Sciences, Nanjing 210014, Jiangsu, China.

^3^ School of Food and Biological Engineering, Jiangsu University, Zhenjiang 212013, Jiangsu, China.

^4^ School of Agricultural Biotechnology, Seoul National University, Seoul 08826, Republic of Korea.

^5^ Food Toxicology and Contaminants Department, National Research Centre, Giza 12622, Egypt.

*For correspondence. Email guqin@njau.edu.cn; Tel. & Fax (+86) 025 84395268.

**Figure S1 FvAtg4 and FvAtg8 are conserved in yeasts and other filamentous fungi.** (a)Alignments of amino acid sequences of Atg4 orthologs from *F. verticillioides* and other fungal species listed. The Boxshade program was used to highlight identical (black shading) or similar (grey shading) amino acids. The peptidase domain is indicated in black box. (b) Alignments of amino acid sequences of Atg8 orthologs from *F.* *verticillioides* and other fungal species listed. (c) Phylogenetic tree showing the relationships of the deduced amino acid sequences of fungal Atg4s or Atg8s generated using the neighbor-joining method with Mega-X 10.0.2 software.


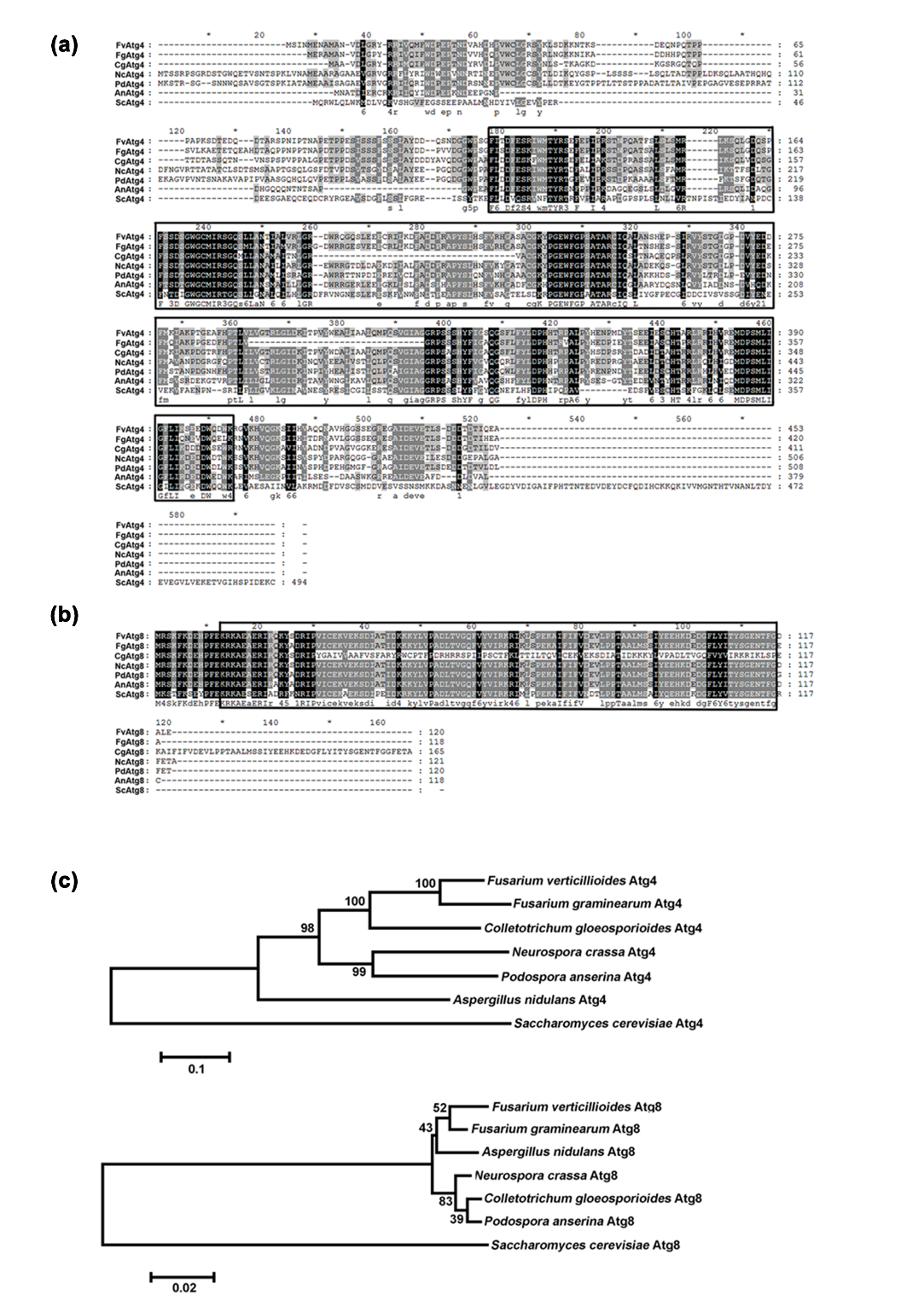


**Figure S2** Schematic representation of the *FvATG4* and *FvATG8* disruption strategy and Southern blotting analysis of the deletion mutants (a). (b) Southern blot analysis of the wild-type strain 7600 and *FvATG4* and *FvATG8* mutants.


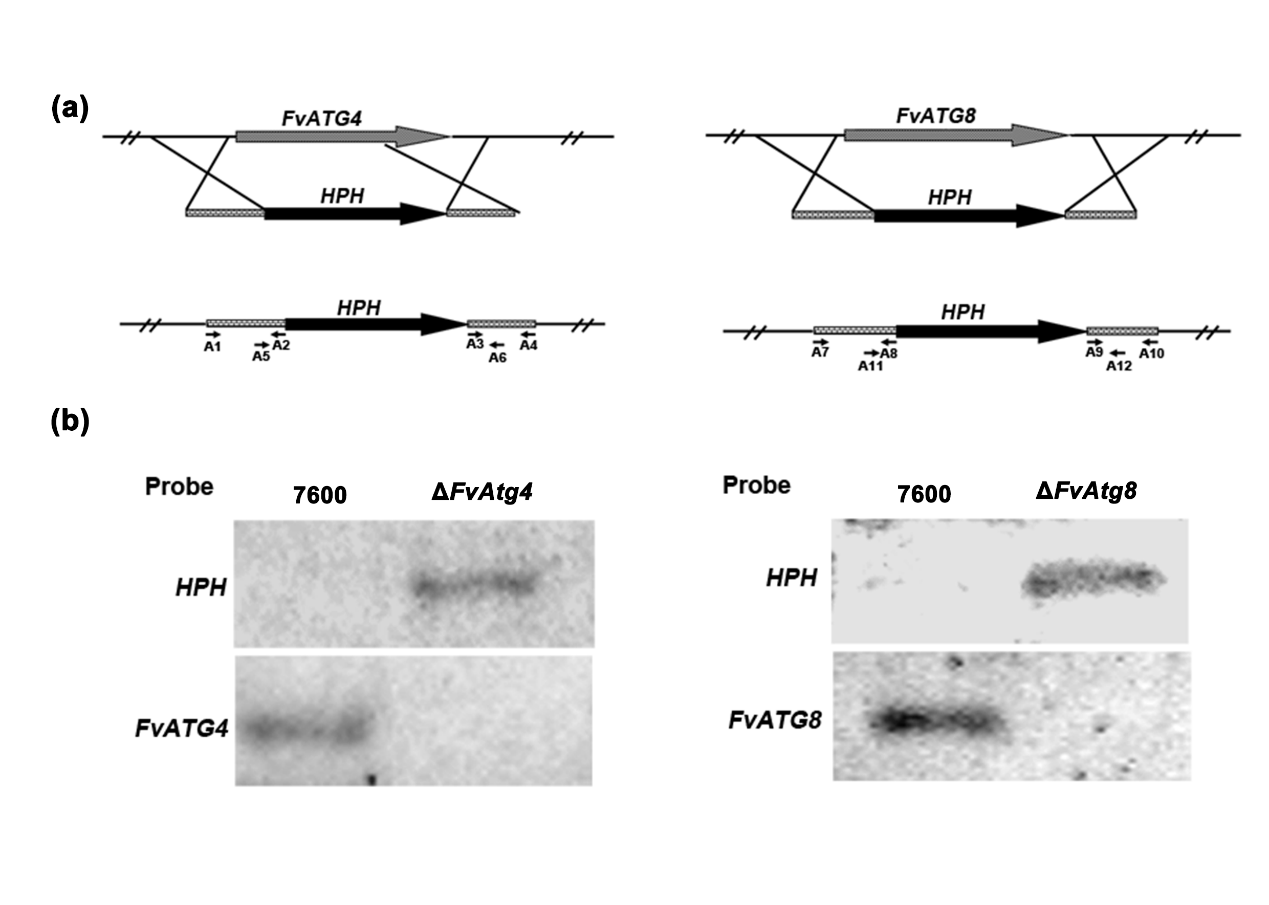


**Figure S3 Rescue of yeast Δ*Atg4* and Δ*Atg8* mutants by the FvATG4 and FvATG8 genes.** (a) RT-PCR analysis of FvATG4 and FvATG8 expression in the *Saccharomyces cerevisiae* strains. Positive control means PCR using the vector of FvATG4-pYES2 or FvATG4-pYES2 as template. Negative control reaction contained ddH2O in place of template. (b) FvATG4 and FvATG8 are able to complement defects in autophagic body formation in the yeast Δ*Atg4* and Δ*Atg8* mutants, respectively, under conditions of nitrogen starvation. The BY4741-derived mutants ΔAtg4 and ΔAtg8 were transformed with pYES2, pYES2-FvATG4, or pYES2-FvATG8. Yeast strains were grown in YPD medium and then transferred to nitrogen-free SD (-N) liquid medium containing 1 mM PMSF. The autophagic bodies were examined under a microscope. Scale bar=5 μm.


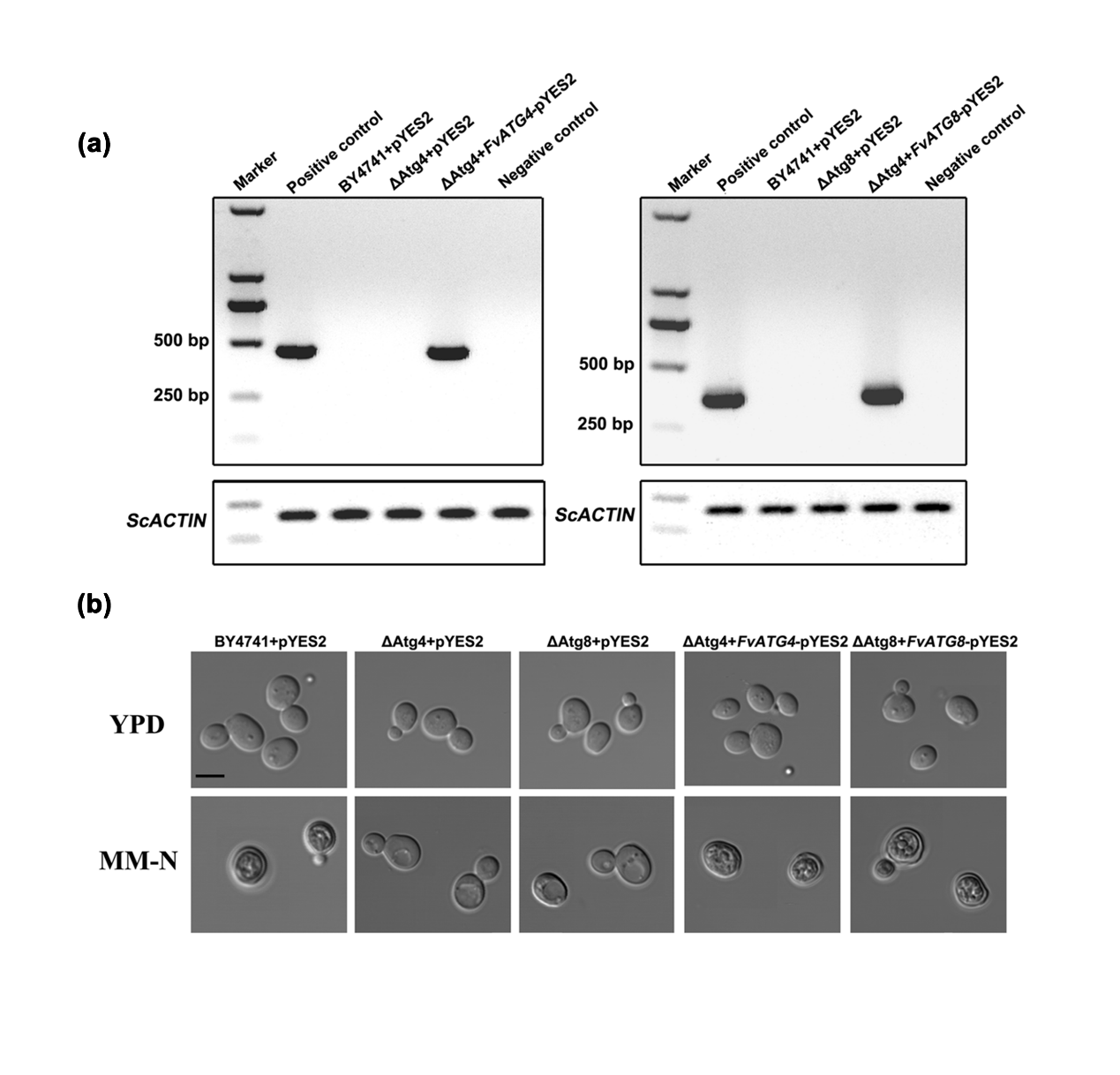


**Figure S4 Effect of FvATG8 deletion on the subcellular localization of FvAtg4 under inducing or non-inducing conditions.** Wild-type 7600 and the ΔFvAtg8 mutant expressing the FvAtg4-GFP fusion protein were grown in liquid YEPD at 25°C and then were shifted to liquid MM-N medium for 4 h. Mycelia were stained with CMAC to observe the vacuoles. BF=Bright Field. Scale bar=10 μm.


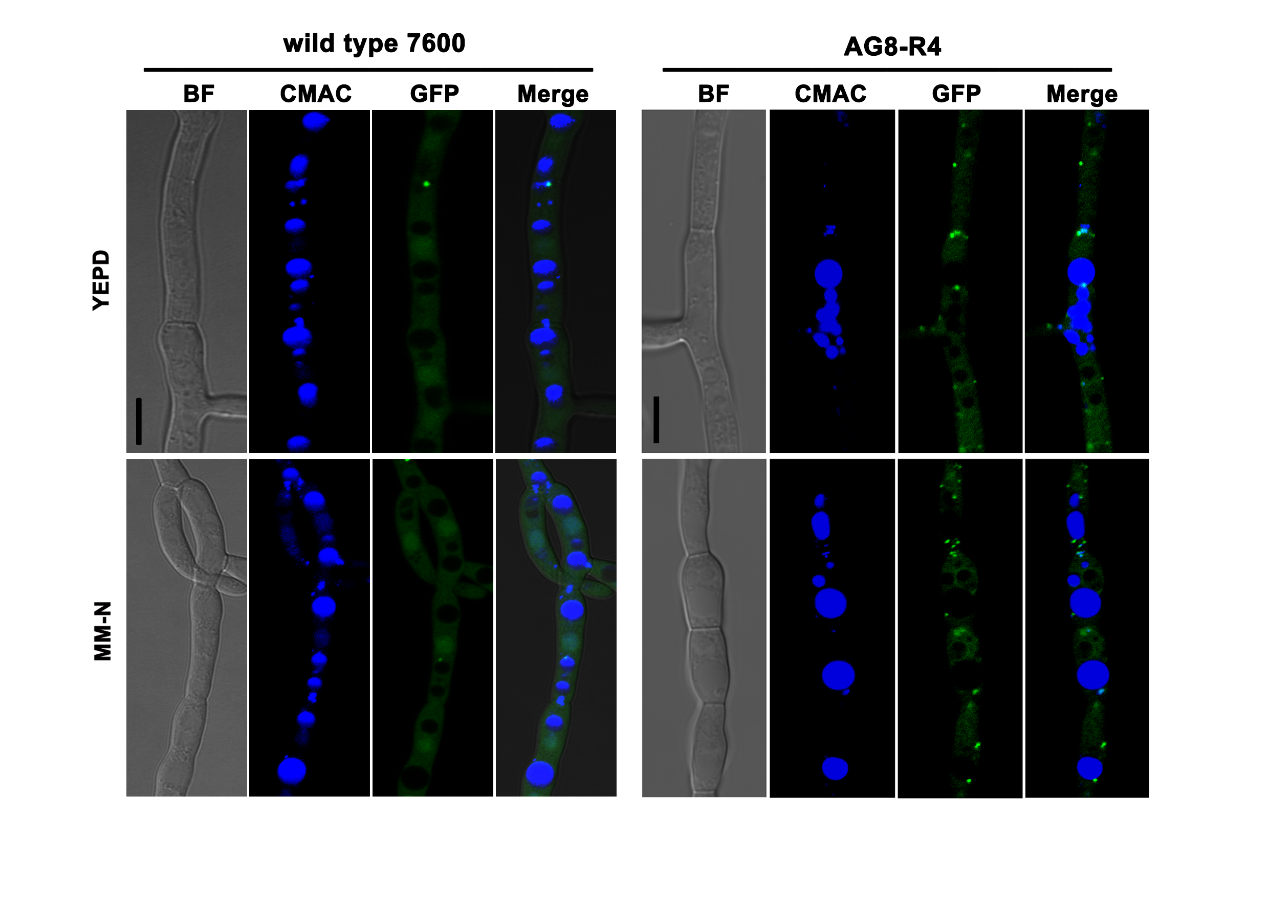


**Figure S5** Assay for aerial hyphal formation in the wild-type strain 7600 and the Δ*FvAtg4*, Δ*FvAtg8*, and Δ*FvAtg4-8* mutants. Each strain was cultured on PDA, CM, and MM medium at 25°C for 4 days. The aerial hyphae of each strain were then observed under a microscope.


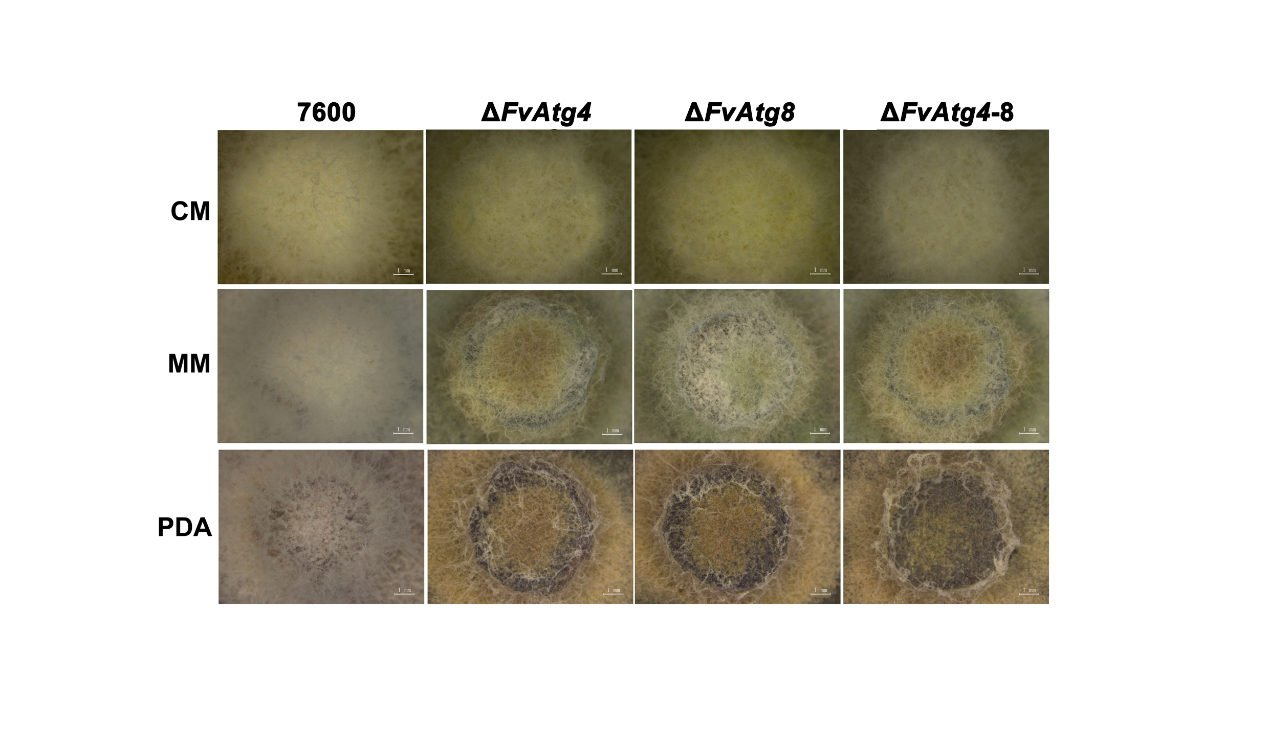


**Figure S6** FT-IR spectra of commercial melanin and the black pigments extracted from the Δ*FvAtg4*, Δ*FvAtg8*, and Δ*FvAtg4-8* mutants of *F. verticillioides*.


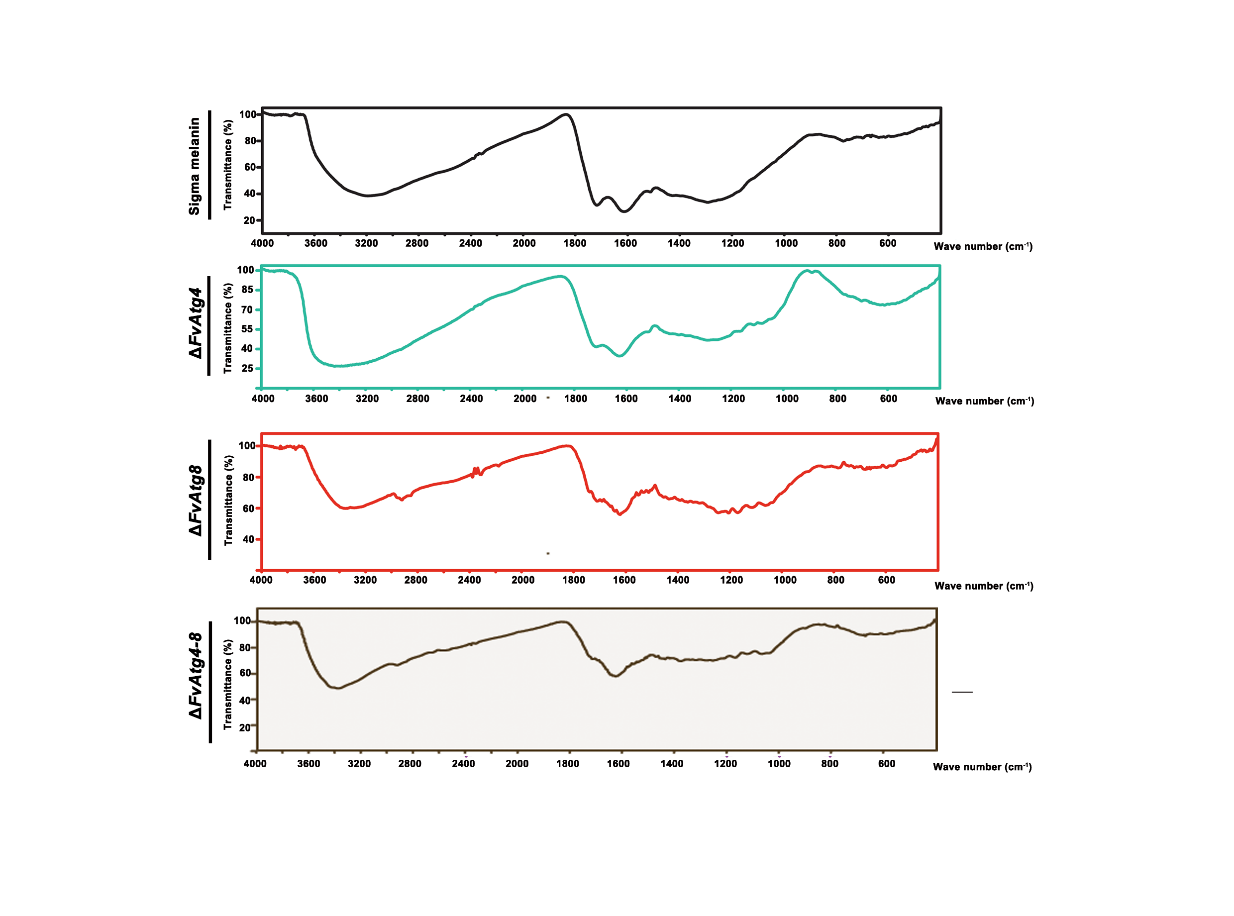


**Figure S7 FvAtg4 and FvAtg8 are involved in bikaverin biosynthesis regulation in *F. verticillioides*.** (a)Effect of *FvBIK1* or *FvBIK3* deletion on the production of red pigment in Δ*FvAtg4* and Δ*FvAtg8* deletion mutants. The double deletion mutants exhibited dramatically reduced bikaverin (red pigment) production in comparison with Δ*FvAtg4* and Δ*FvAtg8* after incubation in potato dextrose broth (PDB) for 10 days. (b) Comparison of wild-type 7600 and the deletion mutants in bikaverin production using HPLC-MS. HPLC-MS peak chromatograms depicting the protonated ion (M+H) + of bikaverin at m/z 383.077 with ∆m/z=5ppm in the mutant strains of Fusarium verticillioides as well as in the wild type.


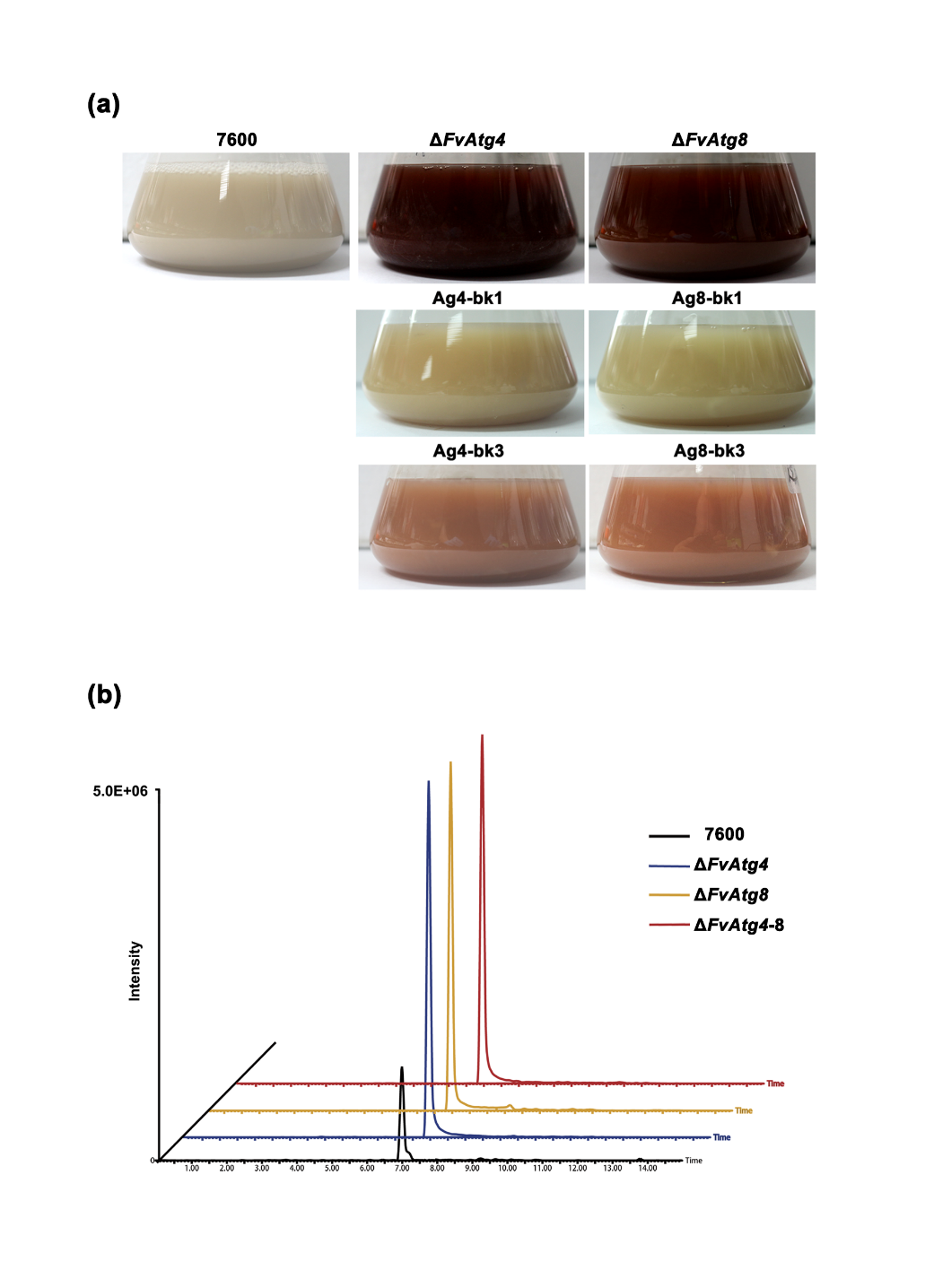


**Figure S8** **Mycelial growth of *F. verticillioides* strains on maize kernel medium and**

**maize stem medium.** (a) *F. verticillioides* strains were grown on maize kernel medium and maize stem medium at 25 °C for 4 days. (b) Mycelial growth was followed by measuring radial extension of the mycelium, Data presented are the mean ± standard deviation from three biological replicates (n = 3). Different letters indicate a significant difference (P < 0.05).


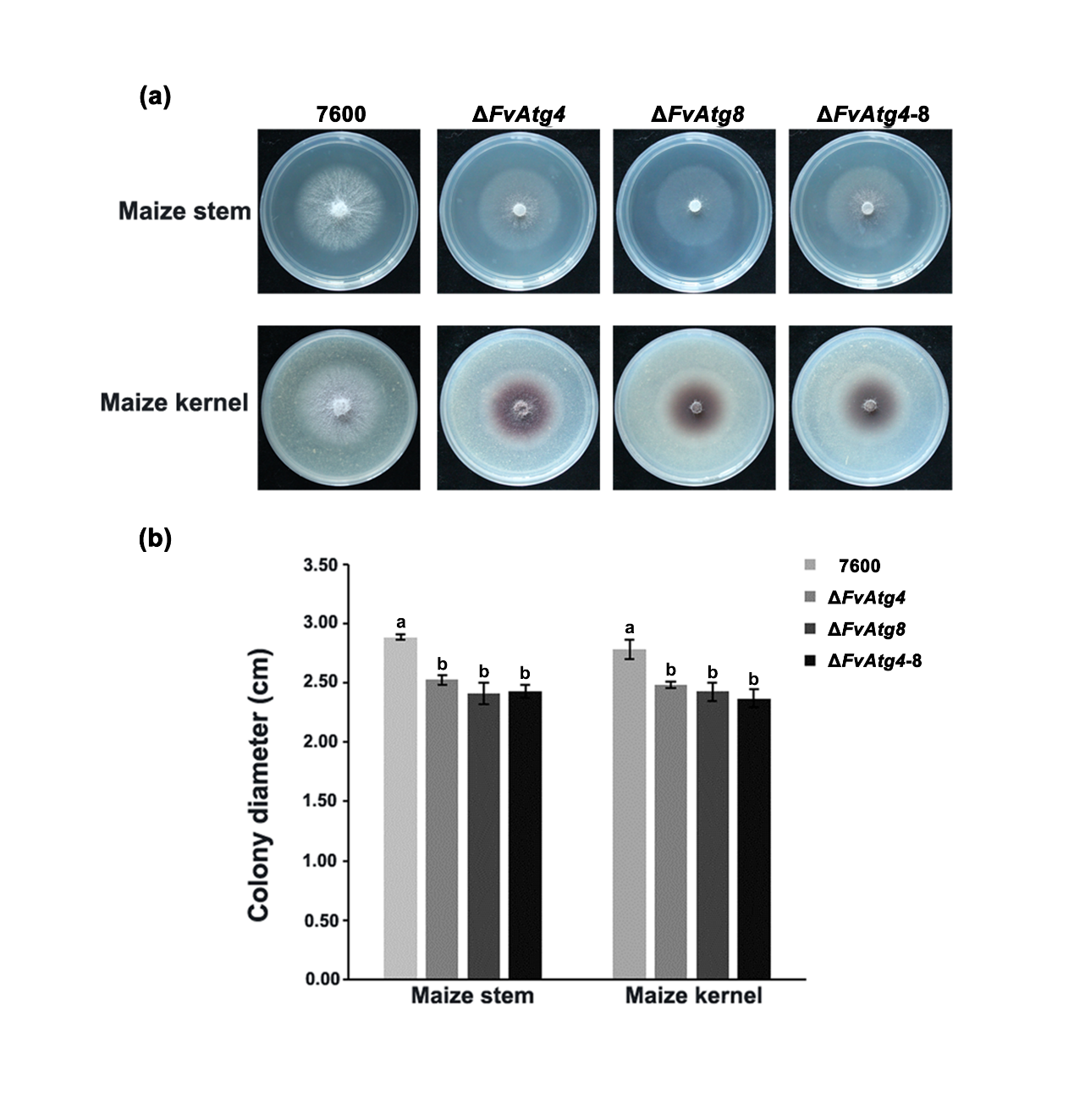


**Figure S9 Assay for asexual and sexual growth in the wild-type, Δ*FvAtg4*, Δ*FvAtg8*, and Δ*FvAtg4-8* mutants.** (a) Macroconidia and microconidia formation was compared between the wild-type (strain 7600) and the mutants. (b) Conidial germination rate of each strain after 2 h or 8 h of incubation. (c) Involvement of FvAtg4 and FvAtg8 in the sexual development of *F. verticillioides.* Perithecial production of the wild-type 7600 strain, ∆*FvAtg4*, ∆*FvAtg8* and ∆*FvAtg4-8*. Black arrows indicate the perithecia.


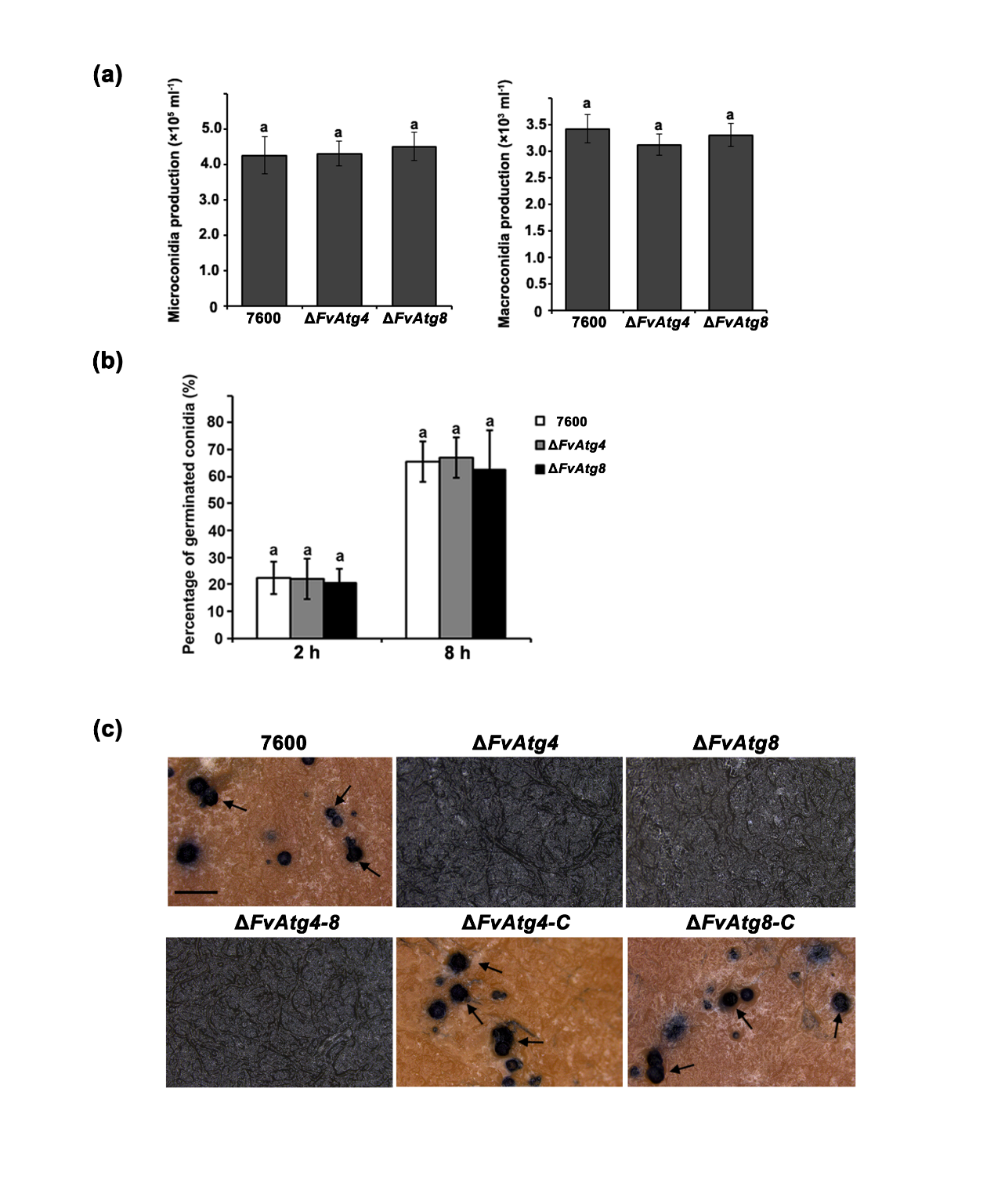


**Figure S10** Relative expression of *FUM* genes in the wild-type strain and the Δ*FvAtg4*, Δ*FvAtg8*, and Δ*FvAtg4-8* mutants in GYAM medium and maize kernels inoculated with each strain on maize kernels (a) and in GYAM medium (b). Line bars in each column denote standard errors of three independent experiments.


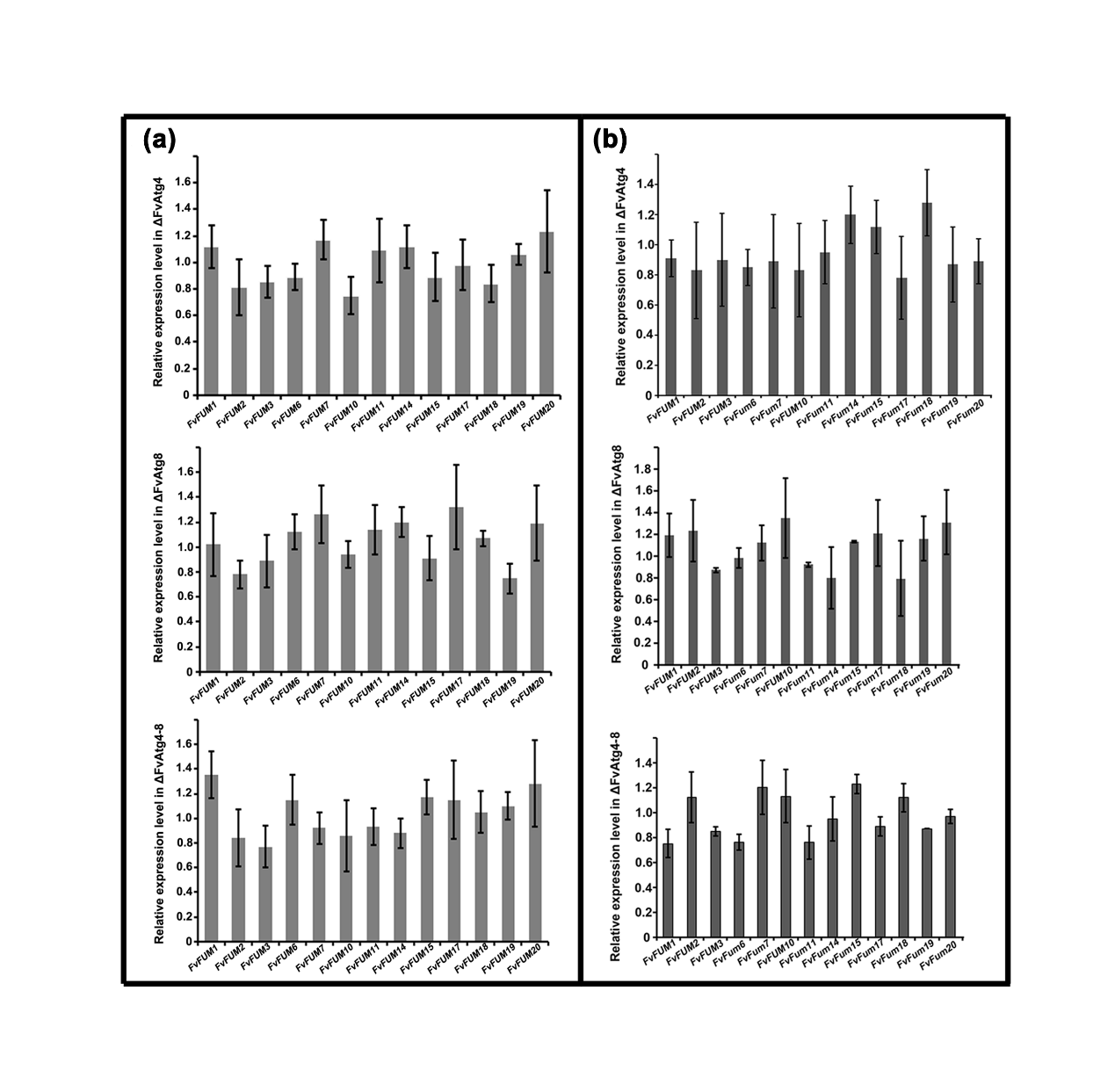


**Table S1. Primers used in this study.**

| Primer | Sequence (5’-3’) | Application |
| --- | --- | --- |
|  |  |  |
| A1 | CATGGCAATATCAAGGAGAGG | A pair of PCR primers for amplification of the upstream sequence of the *FvATG4* gene for construction of the gene deletion vector |
| A2 | CAAAATAGGCATTGATGTGTTGACCTCCGGAAATTGGTGGGAGAATGA |  |
| A3 | CTATCGCCTTCTTGACGAGTTCTTCTGACACGCGATATCAGTTCTAACACA | A pair of PCR primers for amplification of the *FvATG4* downstream fragment for construction of the gene deletion vector |
| A4 | TTGTGGGGAGACTTTTGCTT |  |
| A5 | TCATGTTGCCTCCTTGTTGA | A pair of PCR primers for identification of *FvATG4* deletion mutants |
| A6 | ATCGCCAGTGGAATATACCCT |  |
| A7 | TGCAGTTCGCAGGAAACTTC | A pair of PCR primers for amplification of the upstream sequence of the *FvATG8* gene for construction of the gene deletion vector |
| A8 | CAAAATAGGCATTGATGTGTTGACCTCCTGGGGGTTGGTAAGTTGTAGA |  |
| *HPH*-F | GGAGGTCAACACATCAATGCCTATT | Amplify *HPH* sequence |
| *HPH*-R | CTACTCTATTCCTTTGCCCT |  |
| FvActin-F | TGCTCCTGAGGCTCTCTTCCA | Quantitative real-time PCR primers for analysis of the reference gene actin expression level |
| FvActin-R | AAGCAAGAATAGAACCACCGA |  |
| *FUM1*-F | TGCTGCCCTGTATCACAACCA | Quantitative real-time PCR primers for analysis of *FUM1* gene expression level |
| *FUM1*-R | AATGTGCGCTTGATCCAGTT |  |
| *FUM6*-F | TCTCTTGTTCTTTGGCTGTCG | Quantitative real-time PCR primers for analysis of *FUM6* expression level |
| *FUM6*-R | TCAATTTCTAGCAGCATCGG |  |
| *FUM7-*F | GCATGGAGAGACAAGTTGCA | Quantitative real-time PCR primers for analysis of *FUM7* expression level |
| *FUM7*-R | TCTGATGAAACTGGGCTTCGT |  |
| *FUM21*-F | AGCTTGTCAACCCAGCAGATA | Quantitative real-time PCR primers for analysis of *FUM21* expression level |
| *FUM21*-R | TTCTACCTGATATGCTGTGT |  |
| *FUM*10-F | TTTGGAACCCAATGGCGAT | Quantitative real-time PCR primers for analysis of *FUM10* expression level |
| *FUM10*-R | TTTCGGCAGGGCTGATTTTT |  |
| *FUM11*-F | AAGGGGGGAAGATAGGCACT | Quantitative real-time PCR primers for analysis of *FUM11* expression level |
| *FUM11*-R | ATTACGAGTCTTAGCGAGCGA |  |
| *FUM13*-F | AAACCATGGGATGGTATCAGG | Quantitative real-time PCR primers for analysis of *FUM13* expression level |
| *FUM13*-R | TTTCTGCTGAGCCGACATCAT |  |
| *FUM14*-F | AAGAGGTGCTAAAGACAGCCA | Quantitative real-time PCR primers for analysis of *FUM14* expression level |
| *FUM14*-R | ACTCAGGAGCTGCGACTGATA |  |
| *FUM15*-F | TATCGCCCTGGAAAAGCTTG | Quantitative real-time PCR primers for analysis of *FUM15* expression level |
| *FUM15*-R | TCTGTGGGGTCCATTCAATA |  |
| *FUM17*-F | CTCACGATATCAGTGACCTTT | Quantitative real-time PCR primers for analysis of *FUM17* expression level |
| *FUM17*-R | AGCCATATGATGTTGAGGGT |  |
| *FUM18*-F | TCCTCTTCCTGTTCCTGACGA | Quantitative real-time PCR primers for analysis of *FUM18* expression level |
| *FUM18*-R | TTGTAGTTGAGATTGCTGCCA |  |
| *FUM2*-F | AAGTGCTCGGGGAGCGGGTT | Quantitative real-time PCR primers for analysis of *FUM2* expression level |
| *FUM2*-R | TCGGGGCATAACTCTATATCG |  |
| *FUM3*-F | ACTGATTTCACCGAGGCCAA | Quantitative real-time PCR primers for analysis of *FUM3* expression level |
| *FUM3*-R | AGCGGACCGGAAGCTTCT |  |
| A9 | CTCGTCCGAGGGCAAAGGAATAGAGTAGTGCGTGGAATCGGTTCTATATC | A pair of PCR primers for amplification of the *FvATG8* downstream fragment for construction of the gene deletion vector |
| A10 | GCGAGATATTCGTATTCATGTGT |  |
| A11 | TTCCTCATCCTTCTTCTGCCT | A pair of PCR primers for identification of *FvATG8* deletion mutants |
| A12 | TACAGACGTGTTGCCAGAAAG |  |
| *FvATG4*-AD-F | GCCATGGAGGCCAGTGAATTCATGTCT ATCAACATGGAGAAC | A pair of PCR primers for amplification of the the full cDNA sequence of the *FvATG4* gene to generate the FvATG4-AD construct |
| *FvATG4*-AD-R | ATGCCCACCCGGGTGGAATTCCTAGGCCTCCTGAATGGTAT |  |
| *FvATG4*-BD-F | ATGGCCATGGAGGCCGAATTCATGTCTATCAACATGGAGAACGCC | A pair of PCR primers for amplification of the the full cDNA sequence of the *FvATG4* gene to generate the FvATG4-BD construct |
| *FvATG4*-BD-R | TCGACGGATCCCCGGGAATTCCTAGGCCTCCTGAATGGTATCCG |  |
| *FvATG8*-AD-F | GCCATGGAGGCCAGTGAATTCATGCGCAGCAAGTTCAAGGA | A pair of PCR primers for amplification of the the full cDNA sequence of the *FvATG8* gene to generate the *FvATG8*-AD construct |
| *FvATG8*-AD-R | ATGCCCACCCGGGTGGAATTCTTACTCCAAAGCATCACCGAAGG |  |
| *FvATG8*-BD-F | ATGGCCATGGAGGCCGAATTCATGCGCAGCAAGTTCAAGGACG | A pair of PCR primers for amplification of the full cDNA sequence of the *FvATG8* gene to construct the *FvATG8*-BD vector |
| *FvATG8*-BD-R | TCGACGGATCCCCGGGAATTCTTACTCCAAAGCATCACCGAA |  |
| *FvATG4*-PYES2-F | TCACACTGGCGGCCGCTCGAGATGTCTATCAACATGGAGAACGCC | A pair of PCR primers for amplification of the full cDNA sequence of the *FvATG4* gene to construct the *FvATG4*-pYES2 vector |
| *FvATG4*-PYES2-R | CCCTCTAGATGCATGCTCGAGCTAGGCCTCCTGAATGGTATCCG |  |
| *FvATG8*-PYES2-F | TCACACTGGCGGCCGCTCGAGATGCGCAGCAAGTTCAAGGA | A pair of PCR primers for amplification of the full cDNA sequence of the *FvATG8* gene to construct the *FvATG8*-pYES2 vector |
| *FvATG8*-PYES2-R | CCCTCTAGATGCATGCTCGAGTTACTCCAAAGCATCACCGAAGG |  |
| FvATG4-FLAG-F | CTATAGGGCGAATTGGGTACTCAAATTGGTTGAAAGGTGTTACAGATCTTT | A pair of PCR primers to amplify *FvATG4* fragment used for construction of the *FvATG4*-FLAG vector |
| FvATG4-FLAG-R | CTTTATAATCACCGTCATGGTCTTTGTAGTCGGCCTCCTGAATGGTATCCG |  |
| FvATG4-RFP-F | CTATAGGGCGAATTGGGTACTCAAATTGGTTGAAAGGTGTTACAGATCTTT | A pair of PCR primers to amplify *FvATG4* fragment used for construction of the *FvATG4*-RFP vector |
| FvATG4 -RFP-R | GAACTCCTTGATGACGTCCTCGGAGGAGGCGGCCTCCTGAATGGTATCCG |  |
| FvATG4-F | TGATTGCAGCACTTCAGATGC | Quantitative real-time PCR primers for analysis of *FvATG4* expression level |
| FvATG4-R | AATCGACTTGCCTTGGACAT |  |
| FvATG8-F | GTCATTTGCGAAAAGGTCGA | Quantitative real-time PCR primers for analysis of *FvATG8* expression level |
| FvATG8-R | CCGTCCTCATCCTTGTGTTC |  |
| *FvATG4*-GFP-F | TTTCGTAGGAACCCAATCTTCAAAATGTCTATCAACATGGAGAACGC | A pair of PCR primers to amplify *FvATG4* fragment used for construction of the *FvATG4*-GFP or *FvATG4*-RFP vector |
| *FvATG4*-GFP-R | CACCACCCCGGTGAACAGCTCCTCGCCCTTGCTCACGGCCTCCTGAATGGTATCCGTAT |  |
| *FvATG8*-Promoter-F | AGTGGCCAGCAGAGAAACTTT | A pair of PCR primers to amplify the promoter of *FvATG8* for construction of the GFP- *FvATG8* vector |
| *FvATG8*-Promoter-R | CTCCTCGCCCTTGCTCACATTGACGGTGGGGGTTGGTAA |  |
| *FvATG4*-Promoter-F | ACTCACTATAGGGCGAATTGGGTACTCAAATTGGTTGAAAGGTGTTACAGATCTTT | A pair of PCR primers to amplify the promoter of *FvATG4* for construction of the *FvATG4* promoter-GFP vector |
| *FvATG4*-Promoter-R | CTCCTCGCCCTTGCTCACGGCCTCCTGAATGGTATCCG |  |
| GFP-F | ATGGTGAGCAAGGGCGAGGA | A pair of PCR primers to amplify *GFP* fragment used for construction of the GFP- *FvATG8* vector |
| GFP-R | CTTGTACAGCTCGTCCATGC |  |
| GFP-*FvATG8-*F | GCATGGACGAGCTGTACAAGATGCGCAGCAAGTTCAAGGA | A pair of PCR primers to amplify *FvATG8* fragment used for construction of the GFP- *FvATG8* vector |
| GFP-*FvATG8*-R | TTACTCCAAAGCATCACCGA |  |
| A13 | AGCATTGGCGAACTCACATGA | A pair of PCR primers to analyze the expression of *FvATG4* in *Saccharomyces cerevisiae* strains |
| A14 | CTCCTCACTCCGAATCAGA |  |
| A15 | ATGCGCAGCAAGTTCAAGGAC | A pair of PCR primers to analyze the expression of *FvATG8* in *Saccharomyces cerevisiae* strains |
| A16 | CCGTCCTCATCCTTGTGTTCC |  |
| A17 | CTTTCTGCTGCGCCGTATTT | A pair of PCR primers for amplification of the upstream sequence of the *FvBIK1* gene for construction of the gene deletion vector |
| A18 | CAAAATAGGCATTGATGTGTTGACCTCC TGAAAGCTGTAACAAAGGTGCA |  |
| A19 | CTCGTCCGAGGGCAAAGGAATAGAGTAG CGGAGGGTGAGCTTCTTTCT | A pair of PCR primers for amplification of the downstream sequence of the *FvBIK1* gene for construction of the gene deletion vector |
| A20 | AGCCATCTTGATAGCACCGA |  |
| A21 | ACGTTCCATTGCAGTCACAA | A pair of PCR primers for identification of *FvBIK1* deletion mutants |
| A22 | ACAGAGGAAGATGGCATCGT |  |
| FvBIK1-F | CCTTCAGGCCCTTACCATCT | Quantitative real-time PCR primers for analysis of *FvBIK1* expression level |
| FvBIK1-R | GAGCTTCCTCGCCCATCTC |  |
| FvBIK2-F | CGCACAGCCAGCTACTACA | Quantitative real-time PCR primers for analysis of *FvBIK2* expression level |
| FvBIK2-R | CCCTCGCTTCATCAACAGC |  |
| FvBIK3-F | CCCGATCTCACCTTTATCATCC | Quantitative real-time PCR primers for analysis of *FvBIK3* expression level |
| FvBIK3-R | CAGTTGTGAAAGATTTGGCGC |  |
| A23 | AGCTTTGAGTCAATGTGTGTG | A pair of PCR primers for amplification of the upstream sequence of the *FvBIK3* gene for construction of the gene deletion vector |
| A24 | CAAAATAGGCATTGATGTGTTGACCTCC GAGCCGCGATGATTCAGTG |  |
| A25 | CTCGTCCGAGGGCAAAGGAATAGAGTAG AGCAGAATCGAAAAGAACCGT | A pair of PCR primers for amplification of the downstream sequence of the *FvBIK3* gene for construction of the gene deletion vector |
| A26 | ACGACGTACTGGATGCCA |  |
| A27 | TTCTCAAACATCAGCGCCAG | A pair of PCR primers for identification of *FvBIK3* deletion mutants |
| A28 | ATGGAGGGTGTCAGTCAAGG |  |
| FvBIK4-F | CTGCTGCCGTGTTAGTCAA | Quantitative real-time PCR primers for analysis of *FvBIK4* expression level |
| FvBIK4-R | TCAGGAACATGAAGCTTGGC |  |
| FvBIK5-F | CAAGATGAGACTCGCCTTGC | Quantitative real-time PCR primers for analysis of *FvBIK5* expression level |
| FvBIK5-R | CGGTGTCTGCTGCAAAGG |  |
| FvBIK6-F | CCTGTTCCTGAAGCGAGACT | Quantitative real-time PCR primers for analysis of *FvBIK6* expression level |
| FvBIK6-R | TGAAGCCAACCCCTGTGAG |  |

**Table S2. Functional groups of melanin-like pigment produced by**

***F. verticillioides* and commercial melanin obtained from Fourier Transform Infrared spectroscopy.**

| Assignment | Wave number (cm^-1^) | | | | Reference |
| --- | --- | --- | --- | --- | --- |
|  | **ΔFvAtg4** | **ΔFvAtg8** | **ΔFvAtg4-8** | **Commercial**  **melanin** |  |
| -COOH | 1715 | 1709 | 1712 | 1717 | (Kannan and Ganjewala., 2009) |
| Aromatic ring C=C and C=O, stretching | 1626 | 1621 | 1623 | 1611 | (Kannan and Ganjewala., 2009) |
| -COOH | 1517 | 1511 |  |  | (Kannan and Ganjewala., 2009) |
| C-H, bonding | 1433 | 1433 | 1435 | 1428 | (Coates., 2006) |
| -COO, symmetric stretching | 1399 | 1361 | 1373 |  | (Magarelli et al., 2010) |
| C-H, deformation | 1290 |  | 1252 | 1292 | (Kolczyński et al., 1992) |
| C=O | 1167 | 1204 | 1165 |  | (Kannan and Ganjewala., 2009) |
| C-O, close to aromatic ring | 1081 | 1063 | 1063 |  | (Kolczyński et al., 1992) |
| N-H, O-H, bending | 699 | 741 | 668 | 753 | (Coates., 2006) |

Reference:

Kannan, P., Ganjewala, D. (2009) Preliminary characterization of melanin isolated from fruits and seeds of Nyctanthes arbor-tristis. J Sci Res 1: 655–61.

Coates, J. (2006) Interpretation of infrared spectra, a practical approach. In Mayers RA, editor. Encyclopedia of Analytical Chemistry. Chichester: Jon Wiley & Sons Ltd pp. 10815–10837.

Magarelli, M., Passamonti, P., Renieri, C. (2010) Purification, characterization and analysis of sepia melanin from commercial sepia ink (Sepia officinalis). Rev CES Med Vet Zootech 5: 18–28.

Kolczyński-Szafraniec, U., Bilińska, B. (1992) Infrared studies of natural pheomelanins. Curr Topics Biophys 16: 77–80.
